# Nanofibrillar Cellulose Hydrogels with Anionic Surface Modifications for Modulating Macrophage Phenotype in 3D Culture

**DOI:** 10.1021/acsami.5c06549

**Published:** 2025-07-03

**Authors:** Maria Heilala, Rita Turpin, Nikolaos Pahimanolis, Nonappa, Olli Ikkala, Juha Klefström, Pauliina M. Munne

**Affiliations:** † Department of Applied Physics, Aalto University. P.O. Box 15100, FI-00076 Aalto, Espoo Finland; ‡ Cancer Cell Circuitry Laboratory, Translational Cancer Medicine, Medical Faculty, University of Helsinki. P.O. Box 63, Haartmaninkatu 8, FI-00014 Helsinki, Finland; § Oy Keskuslaboratorio-Centrallaboratorium Ab, Tekniikantie 2, FI-02150 Espoo, Finland; ∥ Faculty of Engineering and Natural Sciences, Tampere University. P.O. Box 541, FI-33720 Tampere, Finland; ⊥ Finnish Cancer Institute, FI-00290 Helsinki, Finland; # FICAN South, Helsinki University Hospital, FI-00290, HUS, Helsinki, Finland; ∇ Department of Cell & Tissue Biology, Universityof California, San Francisco, 513 Parnassus Avenue, UCSF Campus Box 0512, San Francisco, California 94143, United States

**Keywords:** macrophages, 3D cell culture, hydrogels, nanocelluloses, macrophage polarization

## Abstract

Anti-inflammatory M2 macrophages are highly relevant in various physiological processes ranging from tissue regeneration to cancer progression. However, conventional two-dimensional (2D) *in vitro* cell cultures limit our understanding of macrophage phenotypes and how they can be modulated for immunotherapeutic approaches. Moreover, there is a growing demand for scalable, animal-free hydrogels to replace animal-derived materials in three-dimensional (3D) *in vitro* models. In this study, we explore hydrogels based on plant-derived nanofibrillar cellulose (NFC), also known as cellulose nanofibrils (CNFs) or microfibrillated cellulose (MFC), for generating 3D *in vitro* models of M2-like macrophages from human blood monocytes. Notably, flow cytometry analysis shows that cells cultured in 3D phosphorylated NFC hydrogels show enhanced expression of the M2 macrophage marker CD206 compared to cells cultured in other negatively charged hydrogels prepared from native NFC or NFCs with carboxylate or sulfate modifications. Furthermore, the upregulation of CD206 expression in 3D phosphorylated NFC is comparable to the induction of CD206 in interleukin 4 (IL-4)-differentiated M2a macrophages. In addition, the cells in the phosphorylated NFC hydrogel show a differential cytokine profile compared to 2D cultured cells, secreting similar levels of tumor necrosis factor α (TNF-α), but 2.6-fold higher amounts of IL-1β and 1.2-fold higher amounts of IL-10. The results suggest that the conversion of monocytes to M2-like macrophages can be controlled by the phosphorylation of NFC, a strategy which does not require the addition of polarization factors like growth factors and cytokines conventionally used to generate macrophages *in vitro*. The findings highlight the importance of surface chemistry in matrix-guided macrophage polarization, paving the way for xeno-free yet bioactive 3D macrophage culture scaffolds for immunological research.

## Graphical abstract

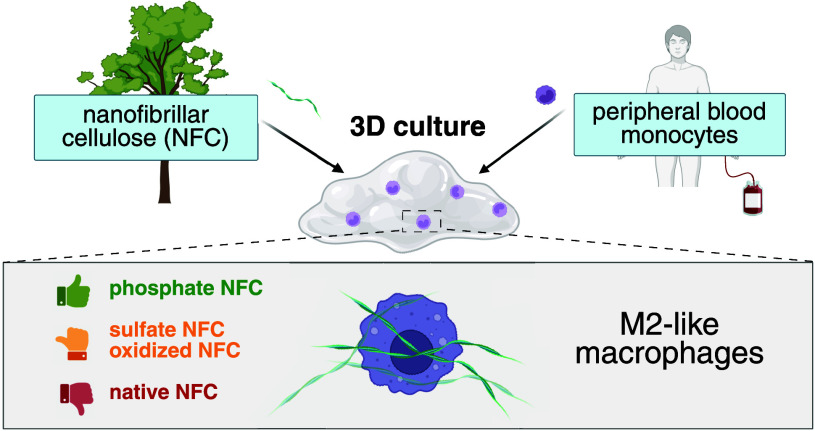

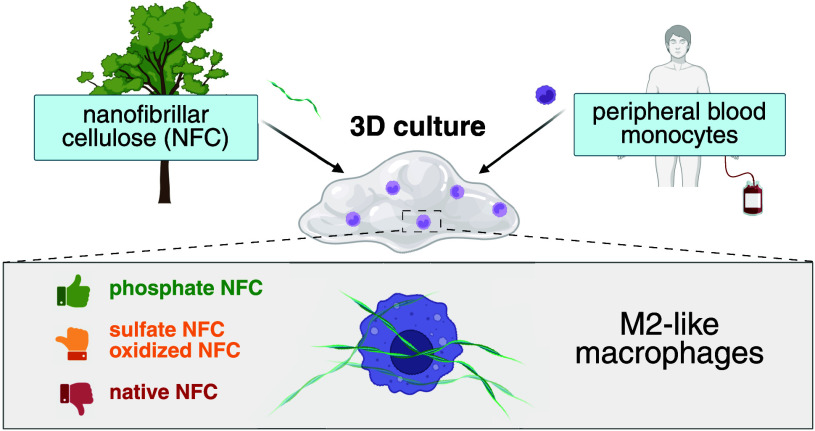

## 1 Introduction

Macrophages are innate immune cells that maintain tissue homeostasis by facilitating processes such as angiogenesis, tissue remodeling, and host defense.^1^ Based on the expression of surface markers and secretion profiles, macrophages are commonly categorized into proinflammatory M1 or anti-inflammatory M2 phenotypes.^2^ The dysregulation of macrophage phenotypes is implicated in various pathophysiological conditions, including inflammatory disorders and cancer.^1^ For example, tumor-associated macrophages (TAMs) resembling immunosuppressive M2 macrophages are associated with poor cancer prognosis and tumor drug resistance.^3,4^ In the context of biomaterials, insufficient M2 macrophage recruitment can lead to excessive inflammation and poor implant integration.^5,6^ To improve cancer immunotherapy and tissue engineering outcomes, *in vitro* models of M2-like macrophages are crucial for understanding the mechanisms that regulate macrophage phenotypes and functions.

Due to challenges in obtaining sufficient numbers of tissue-resident macrophages, *in vitro* studies on macrophage function typically rely on cell lines or macrophages derived from human peripheral blood monocytes.^7^ Both of these cell models can be cultured on standard two-dimensional (2D) tissue culture plastic and polarized to M1 or M2 macrophages by using soluble factors. *In vitro* polarization toward the M2 phenotype is mainly achieved through biochemical stimulation, for example using interleukin (IL)-4, IL-10, or glucocorticoids.^8^ Depending on the activation stimulus, M2 macrophages can be further divided into the M2a, M2b, M2c, and M2d subtypes.^9^ The physicochemical properties of the 2D culture substrate, such as stiffness, surface chemistry, and topography, have also been found to guide macrophage polarization.^10–14^ However, culturing monocytes on 2D substrates or chemically inducing their polarization into M2 macrophages overlooks the *in vivo* conditions, where the macrophage phenotype is influenced by the physical and chemical cues in their three-dimensional (3D) extracellular environment.^15–17^ Moreover, it is recognized that macrophages in tissues can adopt versatile phenotypes that are easily oversimplified in 2D culture.^18^ Due to the phenotypic heterogeneity, it is appropriate to classify macrophages as M1-like/M2-like rather than considering them as opposite ends of the polarization spectrum.

To mimic the 3D environment *in vitro*, cells can be cultured inside hydrogel-based 3D scaffolds. Hydrogels can mimic the key properties of the extracellular matrix (ECM), including high water content, fibrillar architecture, and mechanical support.^19^ For example, 3D hydrogels prepared from a collagen-based matrix and decellurized porcine lymph node ECM have been shown to promote the M2-like phenotype in THP-1 cells and murine bone marrow-derived macrophages, respectively, when compared to the 2D culture setup.^20,21^ However, such animal-derived hydrogels have several drawbacks, such as ethical concerns regarding animal use, batch-to-batch variation, and limited control over the physicochemical properties. Alternatively, polysaccharides represent ethical and easily modifiable source materials for 3D hydrogels. While nonplant-derived polysaccharides, like chemically modified hyaluronan,^22^ have been used as 3D scaffolds for the generation of M2-like macrophages, they are not as readily available as plant-derived polysaccharides. The most abundant polysaccharide on Earth is cellulose, which is a cell wall constituent in all higher plants.^23^ The macroscopic cellulose fibers can be mechanically processed directly into nanofibrils or chemically pretreated to introduce various surface modifications through the hydroxyl (OH) groups.^24,25^ The resulting nanofibrillar celluloses (NFCs) are typically a few to tens of nanometers in diameter but up to several micrometers in length.^26^ As a result of fibril entanglement and retention of large amounts of water, NFCs readily form shear-thinning hydrogels.^25^ NFC gels are temperature-stable and resist degradation by mammalian enzymes, conferring stability during culture. Importantly, in contrast to matrices isolated from animal sources, plant-based NFC gels do not contain unspecified animal-derived components, like contaminating growth factors, that might interfere with cellular responses.^27^

NFC gels have already been used for 3D culture of various cell types, such as liver cancer cells, breast cancer cells, stem cells, and fibroblasts.^28–32^ While commonly used NFC gels are based on native (chemically unmodified) NFC or 2,2,6,6-tetramethylpiperidine-1-oxyl radical (TEMPO)-oxidized NFC, phosphorylation and sulfation represent more recent strategies in NFC modification.^24^ Such modifications are known to modulate the bioactivity of polysaccharides and might impart advantageous properties for 3D immune cell culture scaffolds.^33^ Generally, NFC is not considered to trigger inflammatory responses in monocytes/macrophages *in vitro*, although some studies have suggested the anti-inflammatory potential of certain NFC modifications.^34–39^ However, interactions between NFCs and immune cells have been mainly assessed using dilute NFC fibril suspensions or 2D NFC films.^34–40^ As such, the findings might not be directly generalizable to the immunomodulation occurring when the cells are encapsulated within the 3D ECM-like environment of NFC gels. Therefore, the applicability of surface-modified NFC gels in the 3D culture of M2-like macrophages warrants further exploration.

In this work, we show the potential of surface-modified NFC hydrogels as 3D culture scaffolds for monocytes/macrophages. We use monocytes isolated from human peripheral blood mononuclear cells (PBMCs), an easily available primary immune cell source that captures donor-to-donor variation not represented by cell lines. Instead of simply trying to replicate the biochemical composition of the ECM, our aim is to provide cells with a 3D matrix environment and to induce macrophage polarization to M2-like phenotypes through chemical modifications of the NFCs. Specifically, we focus on (1) characterizing physicochemical properties of NFC gels, (2) assessing the compatibility of NFC gels with primary monocytes in 3D culture, and (3) establishing whether specific surface modifications of NFC gels affect macrophage polarization toward M2-like phenotypes. To achieve this, NFC gels with different surface properties, including unmodified, oxidized, phosphorylated, and sulfated NFC, are first assessed for their immunomodulatory activities by performing gene expression analysis on PBMCs cultured inside the gels. This is followed by monocyte-only cultures, where monocyte viability and differentiation into M2-like macrophages are evaluated. The cytokine release profile of cells cultured in the NFC gel that most efficiently promotes the M2-like macrophage phenotype is then determined. Finally, the role of proteins in mediating macrophage polarization in this culture system is investigated. The study design, culture setups, and methodologies used to evaluate macrophage phenotypes are summarized in Scheme 1.

**Scheme 1:**
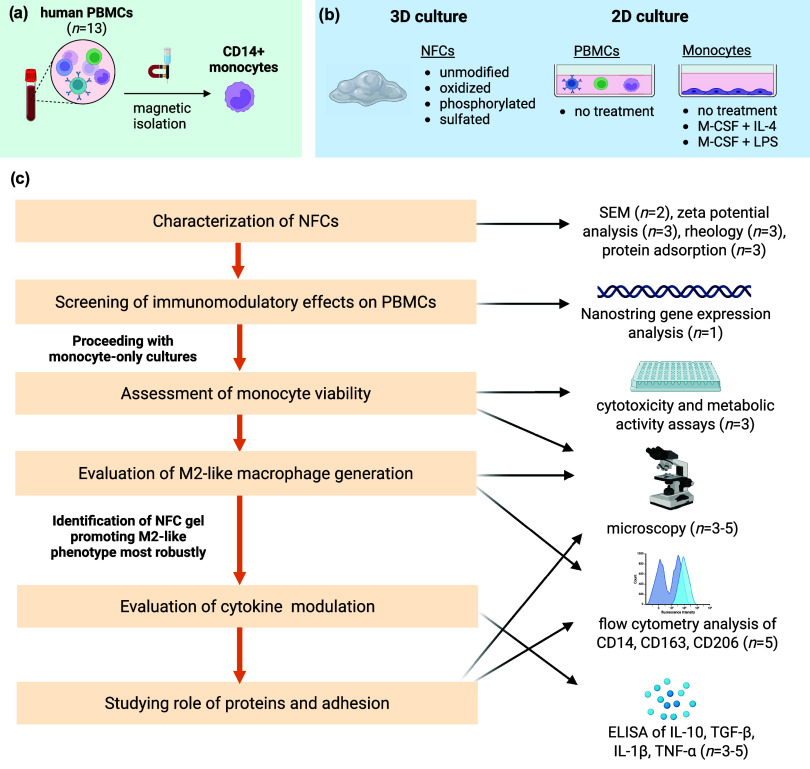
Schematic Summarizing the Study Design^a^

a (a) PBMCs and monocytes were used for the cell culture experiments. (b) Representation of the cell culture setups used in the study. (c) Flowchart of the experimental steps and the analytical methods used to characterize NFCs and cell phenotypes. For each method, *n* denotes the number of independent samples or donors analyzed.

## 2 Materials and Methods

### 2.1 Cellulose Materials

0.5% (w/v) phosphate- and 1% (w/v) sulfate-modified nanofibrillar cellulose (NFC) gels were provided by KCL Oy Keskuslaboratorio-Centrallaboratorium Ab (Finland). The degree of derivatization was 0.84 mmol/g, corresponding to a degree of substitution (DS, the average number of substituted hydroxyl groups per anhydrous glucose unit) of 0.14. These NFCs were autoclaved for sterility at 121 °C for 15 min. The 1.5% (w/v) native, chemically unmodified (GrowDex) and 1% (w/v) carboxylate-modified (GrowDex-T)^31^ NFC gels were purchased from UPM Kymmene Oyj (Finland). All NFC gels were derived from birch. The DS of UPM’s carboxylate NFC is typically between 0.12 and 0.2 ^41^ and thus in a similar range as the phosphate and sulfate NFC gels. The chemical structures of the NFCs used in this study are presented in Figure 1. Before cell culture, 10× PBS and NFC gels were mixed at a 1:19 (v/v) ratio to adjust the osmolarity closer to cells’ needs but to avoid excess dilution of the gels. The use of a positive-displacement pipet is recommended for accurate pipetting of the viscous NFC gels.

**Figure 1:**
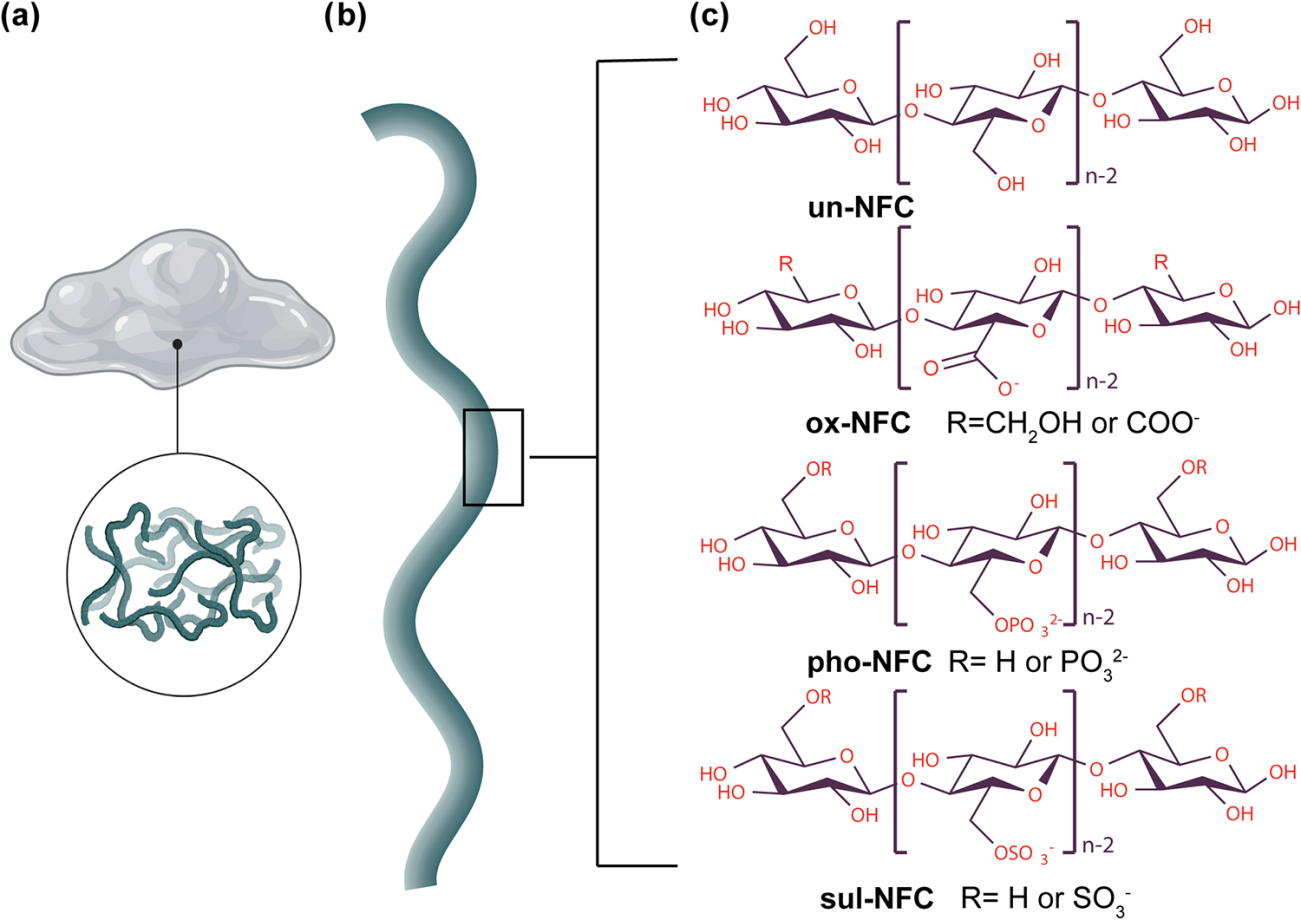
Chemical structures of nanofibrillar celluloses used in this study. (a) Schematic representation of the NFC hydrogel and its 3D fibrillar network. (b) Schematic representation of a single NFC fibril. (c) Chemical structures of unmodified and chemically modified NFCs showing the repeat units. For clarity, only the cellulose polymer is shown, although wood-derived NFC fibrils also contain hemicellulose. R marks the primary site for the chemical substitution reaction (the C6 hydroxyl group). It should be noted that substitution mainly occurs on fibril surfaces and not in all internal cellulose chains within the nanofibrils. Abbreviations denote chemically unmodified NFC (un-NFC), oxidized NFC (ox-NFC), phosphorylated NFC (pho-NFC), and sulfated NFC (sul-NFC).

### 2.2 ζ-Potential

The surface charge of NFC fibrils was assessed with a Zetasizer Nano ZS90 analyzer (Malvern Instruments) using a folded capillary cell (Malvern Instruments) or a dip cell (Malvern Instruments). The electrophoretic mobility of the samples was used to derive the ζ-potential using the Smoluchowski equation. Before measurement, NFCs were diluted to 0.01% in Milli-Q water or 0.1× PBS and sonicated for 45 s at 25% amplitude (Branson S-450), and the temperature was equilibrated to 25 °C for 120 s. The measurements were performed on three independent samples with three runs per sample.

### 2.3 Rheological Measurements

Rheological properties of NFC gels were measured with an MCR 302 rheometer (Anton Paar) using a profiled 25 mm parallel plate to prevent sample slippage. Gels were loaded between the plates, and the edges were coated with a thin layer of silicone oil to prevent water evaporation. Gels were presheared at 3 s^–1^ for 30 s and allowed to rest for 5 min to erase sample shear history. A 30 min time sweep at 1% strain and 10 rad/s angular frequency was then carried out at 37 °C, followed by a frequency sweep with 1% strain and an amplitude sweep with an angular frequency of 10 rad/s. The viscoelastic parameters (shear storage modulus *G*′, shear loss modulus *G*″, and phase angle δ) were calculated from the averaged values in the time sweeps. Three independent samples were measured per hydrogel, unless specified otherwise.

### 2.4 Scanning Electron Microscopy (SEM) Imaging

The specimens for SEM imaging were prepared by using the critical point drying method. NFC gels were dehydrated with five exchanges of 100% ethanol, for 1 h each. The final exchange was performed at least overnight. Ethanol was substituted for liquid CO_2_ in a Bal-Tec 030 critical point dryer (Leica Microsystems) by changing the medium multiple times at 5-10 °C. The temperature was raised to 40 °C to bring CO_2_ above the supercritical point and gaseous CO_2_ was released to dry the sample. The dried sample was cut into smaller pieces, mounted onto a carbon adhesive, and coated with 4 nm iridium or gold/palladium in a Leica ACE600 sputter coater (Leica Microsystems). The fibril network morphology was then observed using a field emission scanning electron microscope Sigma VP with a Gemini column (Zeiss) using a 1.5 kV acceleration voltage. The average fibril diameters were obtained by measuring 100 individual fibrils using ImageJ (Fiji)^42^ from two individually prepared specimens. Fibril aggregates were not included in the calculations because their boundaries were not clearly distinguishable, and the possibility that they had formed during the drying process.

### 2.5 Isolation of Peripheral Blood Mononuclear Cells (PBMCs)

PBMCs were isolated with Ficoll-Paque extraction from blood samples obtained from the Finnish Red Cross blood service under ethical permit 15/2023. The blood was diluted 1:1 with a physiological salt solution (MQ water with 0.9% NaCl) and centrifuged in the swing-out rotor at 450*g* for 25 min at 20 °C, selecting acceleration/deceleration rates of 6/0 (no brake). The buffy coats containing the PBMCs were aspirated from the interphase between Ficoll and the medium. The cells were washed twice with sterile PBS containing 4 μg/mL heparin (Sigma-Aldrich) by centrifuging them at 450*g* for 10 min at 4 °C. The PBMCs were frozen with RPMI (50% FBS + 10% DMSO) at −80 °C overnight and then moved to −140 °C. In total, PBMCs from 13 donors were used. Cell culture experiments or monocyte isolation was performed immediately after thawing of the cells.

### 2.6 Monocyte Separation

PBMCs were briefly thawed and suspended into a wash buffer (PBS with 1% heat-inactivated FBS and 2 mM EDTA). The cells were washed by centrifugation at 416*g* for 5 min, resuspended with 200 μL of cold wash buffer, and mixed with 10 μL of CD14 MicroBeads (Miltenyi Biotec). After 15 min of incubation at 4 °C, the cells were washed again and resuspended with 400 μL of cold wash buffer. CD14+ cells were magnetically separated with MS columns (Miltenyi Biotec) according to the manufacturer’s instructions, after which they were immediately used for 2D or 3D culture.

### 2.7 Cell Culture

Cells were cultured in the MammoCult medium (STEMCELL Technologies) supplemented with 10% MammoCult proliferation supplement (STEMCELL Technologies), 2 mM l-glutamine (Thermo Fisher Scientific), 4 μg/mL heparin (Sigma-Aldrich), 20 μg/mL gentamicin (Sigma-Aldrich), 0.1 μg/mL amphotericin B (Biowest), and 1% penicillin/streptomycin (Lonza). For the cultures with reduced protein content, the concentration of the proliferation supplement in the cell culture media was decreased to 1%. For 3D culture, cells were mixed with the NFC gel in a ratio of 1:20 (v/v). 40–50 μL portion of this mixture was pipetted onto a 96-well tissue culture plate, and the medium was carefully added on top. For the 2D culture without any gel, cells were mixed with the medium only (control). The cell-seeding densities are listed in the sections describing each assay. The final volume in all wells was 200 μL. To compare macrophage marker expression and anti-inflammatory cytokine secretion with the cytokine-induced cell phenotype, monocyte differentiation into M2a macrophages was promoted by supplementing 2D cultures with 50 ng/mL macrophage colony-stimulating factor (M-CSF, Miltenyi Biotech) from day 0 and by adding 20 ng/mL IL-4 (Miltenyi Biotech) on day 3. To compare proinflammatory cytokine secretion with the cytokine profile of proinflammatory macrophages, M-CSF-differentiated macrophages in 2D culture were stimulated on day 5 with 100 ng/mL lipopolysaccharide (LPS, from 0111:B4, Sigma-Aldrich) for 24 h. These components were not added to the medium of the cells cultured in the 2D control or in 3D gels. Cells were cultured for 1–6 days in a humidified incubator at 37 °C with atmospheric oxygen and 5% CO_2_, with 100 μL of fresh medium added on day 3.

### 2.8 NanoString Gene Expression Profiling

For a preliminary screening of the effect of different NFC gels on immune cell function, multiplex gene expression profiling was performed on PBMCs from one donor. First, 1 × 10^6^ PBMCs/well were seeded as described above with the exception that a 96-well ultralow attachment plate was used, and the cells were cultured for 48 h. The cell density in the gels equaled 2 × 10^7^ PBMCs/mL gel. For total RNA extraction, the cells were collected by centrifugation and lysed by adding 1 mL of Trizol (Invitrogen) and passing the suspension through a 21G needle. The RNA extraction was performed according to the manufacturer′s instructions until the aqueous phase was obtained, which was then mixed with an equal volume of 70% ethanol. The sample was transferred into a mini-spin column (RNeasy Mini, Qiagen) and processed according to the manufacturer’s instructions. The combination of phenol extraction and column purification led to higher-purity RNA than phenol extraction alone, measured by a NanoDrop ND1000 spectrophotometer (Thermo Fisher). The extracted RNA was treated with DNase and purified with a Clean and Concentrator kit (Zymo) according to the manufacturer’s instructions. NanoString gene expression profiling was then performed at the Biomedicum Functional Genomics Unit (University of Helsinki, Finland). The RNA quantity and quality were assessed with a 4200 TapeStation system (Agilent). After molecular barcoding, 40–60 ng of the total RNA per sample was run on an nCounter SPRINT Profiler system (NanoString Technologies) using the commercial nCounter Human PanCancer Immune Profiling panel with 770 immunology-related genes including 14 internal reference controls (XT-PGX-Huv1, NanoString Technologies). Data was analyzed using nSolver 4.0 software with an nCounter Advanced Analysis 2.0 add-on (NanoString Technologies). The background was subtracted using a threshold of 20 counts, and the raw counts were normalized to positive controls A–E and to 38 of the 40 housekeeping genes included in the panel, excluding two genes with the highest %CV and the lowest count (*POLR2A* and *CC2D1B*).

### 2.9 Cell Viability and Cytotoxicity Assays

CellTox cytotoxicity assay, AlamarBlue viability assay, and CellTiter Glo viability assay were performed to assess the suitability of NFC gels for monocyte culture. Cells from three different donors were used, with cells from each donor seeded at the same time for all three assays. For CellTox and AlamarBlue assays, the cells were seeded into the gels as described in section 2.7, and triplicate wells were pipetted into a black-walled 96-well plate at a density of 2–2.5 × 10^4^ cells/well, corresponding to 5 × 10^5^ cells/mL gel. To estimate the potential background signal coming from the matrix or the cell culture medium, acellular samples were also prepared in triplicate. Cells in 0.5% Triton X-100 in the cell culture medium were used as the cytotoxicity control. The CellTox Green reagent (1:2000, Promega) was added to the cell culture medium and the cells were incubated overnight. The AlamarBlue cell viability reagent (Invitrogen) was added to the same wells with CellTox (10% v/v cell culture medium). The cells were further incubated for 8 h. Twenty-four hours after seeding, the fluorescence of CellTox (ex. 485/em. 520) and AlamarBlue (ex. 544/em. 590–10) was measured with a FLUOstar Omega microplate reader (BMG Labtech). Fluorescence intensities of acellular blanks were subtracted from fluorescence intensities of cell-containing samples, and the values in 3D gel cultures were normalized to the respective donor cultured in 2D. For CellTiter Glo assay, cells were seeded similarly as for CellTox green and AlamarBlue assay, except that a white 96-well tissue culture-treated plate was used. After 24 h, 100 μL of medium was removed from the wells, and the plate was equilibrated to RT for 30 min. 100 μL of the CellTiter Glo 3D reagent (Promega) was added, and the cells were lysed by shaking the plate on an orbital shaker at 750 rpm for 5 min. After 25 min incubation in the dark, luminescence was measured with a FLUOstar Omega microplate reader (BMG Labtech). With CellTiter Glo, the signal from acellular samples was negligible (<1% of the signal from cell-containing samples), making background subtraction unnecessary.

### 2.10 Immunofluorescence Confocal Microscopy

Monocytes were seeded at 1 × 10^5^ cells/well (2 × 10^6^ cells/mL gel) as described above, except that an 18-well μ-Slide (Ibidi) was used, and the final volume in the wells was 140 μL. On day 3, 60 μL of medium was added. On day 6, cells were fixed with 2% paraformaldehyde (PFA) in PBS for 15 min and washed 3× with PBS. For cytoskeletal staining, cells from three different donors were permeabilized 20 min with 0.25% Triton X, washed, and stained with nuclear dye Hoechst 33258 (1:10000, B2883, Sigma-Aldrich) and AlexaFluor 546-conjugated phalloidin (1:400, A22283, Thermo Fisher Scientific) in PBS for 20 min at RT and washed again. For staining monocyte/M2 macrophage markers, fixed cells from four different donors were incubated with 10% normal goat serum in immunofluorescence buffer (7.7 mm NaN_3_, 0.1% BSA, 0.2% Triton, 0.05% Tween20 in PBS) 1 h at RT to prevent nonspecific binding, after which the samples were stained with mouse antihuman CD14-AlexaFluor647 (1:60, 367128, clone 63D3, BioLegend), mouse antihuman CD206-AlexaFluor488 (1:160, 321114, clone 15–2, BioLegend), and Hoechst 33258 in blocking buffer. After 24 h of incubation at 4 °C, the samples were washed with PBS. The cells were imaged with a confocal laser scanning microscope Leica TCS SP8 CARS with a HC PL APO 20× /0.75 or 40× /1.10 water CS2 objective (Leica Microsystems). Brightness and contrast were adjusted in ImageJ for enhanced visibility without altering the underlying pixel values.

### 2.11 Flow Cytometry

Monocytes were seeded in 96-well tissue culture plates as described above, with 1 × 10^5^ cells/well (2 × 10^6^ cells/mL gel). After 6 days in culture, 2D cultured cells were detached from the well plate by washing the wells twice with cold PBS and incubating with macrophage detachment solution (PromoCell) for 40 min at 4 °C and 20 min at RT. For 3D cultures, the supernatant was removed, and each gel was mixed with 100 μL of 5 mg/mL cellulase (Growdase, UPM) diluted in the cell culture medium. The enzymatic dissociation of the gels was carried out in a 37 °C incubator for 1 h. Both 2D and 3D cultured cells were passed through 35 μm cell strainer caps into round-bottom Falcon tubes (Corning) and washed with wash buffer (PBS with 1% heat-inactivated FBS and 2 mM EDTA) for 5 min at 400*g* at 4 °C. The cells were resuspended in cold staining buffer (PBS with 10% heat-inactivated FBS and 2 mM EDTA), transferred to round-bottom 96-well plates, and blocked with anAnti-Hu Fc receptor-binding inhibitor (Invitrogen) to prevent nonspecific binding of antibodies. The cells were labeled by incubating themfor 45 min at 4 °C with mouse antihuman CD14-Pacific blue (301828, clone M5E2, BioLegend), CD163-BV650 (563888, clone GHI/61, BD Biosciences), and CD206-PE (555954, clone 19.2, BD Biosciences) fluorescent antibodies, after which the cells were washed, centrifuged, and resuspended with 90 μL of cold wash buffer. To exclude dead cells from the analysis, 1 μL of SYTOX green (Invitrogen) was added to the cells immediately before running flow cytometry analysis on a NovoCyte Quanteon 4025 flow cytometer (Agilent). A compensation panel to differentiate between distinct populations of cells was created by mixing one drop of UltraComp eBeads Compensation Beads (Thermo Fisher Scientific), with each fluorochrome in separate wells. Unstained cells were used as controls for the background fluorescence. The data were analyzed with FlowJo (v10.8.1, BD Biosciences) to determine the proportion of cells positive for the marker and the shifts in the median fluorescence intensity (MFI). MFI values of 3D cultured cells were normalized to the 2D control to account for differences in absolute values between donors. For evaluating the effect of protein supplementation on macrophage generation, CD206 expression in cells cultured in Mammocult with either 10% or 1% proliferation supplement was compared. For each donor, cells were cultured, processed, and analyzed in parallel, allowing one to directly compare the effect of culture conditions on the cells. In total, cells from nine different donors were cultured and cells from five donors were compared in each flow cytometry experiment.

### 2.12 Enzyme-Linked Immunosorbent Assay (ELISA)

Human DuoSet ELISA kits (R&D Systems) were used to quantify IL-10 (DY217B-05), TGF-β1 (DY240–05), TNF-α (DY210–05), and IL-1β/IL-1F2 (DY201–05) secreted by the monocyte-derived cells during culture in the 3D pho-NFC gel and in 2D culture. The seeding density was 2 × 10^6^ cells/mL gel or 1 × 10^5^ cells/well in 2D controls. The cultures were prepared with cells from nine different donors, and samples from five donors were used for the quantification of each cytokine. For comparison, 2D cultured cells were stimulated with IL-4 or LPS (described in Section 2.7), after which cytokines from five or three donors were measured, respectively. Additionally, a known amount of the ELISA kit’s standard solution was added to the cell culture medium and incubated with acellular NFC gels to check whether the cytokines were released in the supernatant or partially trapped in the NFC matrix. The supernatants from the cultures as well as from acellular samples were collected on day 6 and assayed according to the manufacturer’s instructions. All samples and standards were prepared in duplicate wells. The optical density was measured using a FLUOstar Omega microplate reader (BMG LABTECH) at 450 nm using wavelength correction at 570 nm.

### 2.13 Protein Adsorption Assays

Protein adsorption onto NFCs was studied by mixing 50 μL of 0.5% NFC with 200 μL of the complete Mammocult medium, 100% proliferation supplement, or 100% FBS. Three independent samples were prepared. After 30 min of incubation at 37 °C, NFC was pelleted by 5 min centrifugation at 10000*g*. The remaining protein in the supernatant was quantified using the DC protein assay kit according to the manufacturer’s instructions (Biorad) and by measuring the absorbance of duplicate wells at 690 nm with a FLUOstar Omega microplate reader (BMG Labtech). The NFC pellet was then washed three times with PBS to remove weakly adsorbed proteins. Finally, NFCs were resuspended into 40 μL of PBS and 10 μL of 5× Laemmli sample buffer and incubated for 5 min at 95 °C to solubilize proteins adsorbed to the NFC surface. As controls, protein supplement diluted 1:50 or Mammocult medium diluted 1:5 were denatured similarly. The samples were centrifuged, and the supernatant was loaded onto a 4–20% Mini-PROTEAN TGX stain-free gel (Biorad) with Precision Plus Protein Dual Color Standards (Biorad) as a ladder on each gel. Electrophoretically separated proteins were activated by 5 min of UV exposure and imaged with Gel Doc XR + (Biorad). The images were labeled by the molecular weight positions of the ladder. Densitometric analysis of protein bands was done with ImageJ. For evaluating the NFC surface charge after interaction with medium components, 100 μL of 0.5% NFCs were mixed with 500 μL of Mammocult and incubated for 30 min at 37 °C, after which the samples were diluted 1:10 in Milli-Q-water, sonicated, and the ζ-potential was measured with a Zetasizer instrument identically to other samples (described in Section 2.2).

### 2.14 2D Culture on Phosphate NFC

To evaluate the monocyte interaction with pho-NFC, 1.6–2 × 10^4^ monocytes in 100 μL of media were added onto an 18-well μ-Slide (Ibidi) coated with 50 μL of phosphate NFC or to an uncoated well. After 24 h, the cell morphology was imaged with an Olympus CKX53 microscope. Cells from three different donors were used for the experiment.

### 2.15 Statistical Analysis

The results are expressed as average ± standard deviation. The Kruskal–Wallis test with Dunn’s post hoc test for pairwise comparisons was performed to compare differences between cell populations in flow cytometry (percentage of cells positive for CD14 and CD206 and the non-normalized MFI values) and the cytokine concentrations in ELISA. For comparing the conditions in flow cytometry, the significance values were adjusted by the Bonferroni correction for multiple groups. For secreted cytokines, nonadjusted significance values arereported, as the comparison was made only between two conditions (phosphate NFC and unstimulated control). SPSS 28 (IBM) was used for the statistical analyses, and *p*-values below 0.05 were considered significant.

## 3 Results

### 3.1 Chemical Modification Imparts NFCs with a High Surface Charge

Chemical modifications are known to alter interactions between NFC fibrils, which affect gel properties such as charge, microstructure, and viscoelasticity. To investigate how the physicochemical properties of the matrix influence immune cell-material interactions in 3D culture models, we first characterized the morphology of the NFC network and the surface charge of the NFC fibrils. Hereafter, we refer to native NFC as unmodified NFC (un-NFC, containing OH groups) and to the chemically modified NFC gels as oxidized NFC (ox-NFC, containing COO^–^ groups), phosphorylated NFC (pho-NFC, containing OPO_3_^2–^ groups), and sulfated NFC (sul-NFC, containing OSO_3_^–^ groups) (Figure 1). At physiological pH, the chemically modified NFCs are expected to be deprotonated at one hydroxyl position per substituent, resulting in a net charge of −1 (carboxylate p*K*_a_ ∼ 2.8–3.7, phosphate p*K*_a1_ ∼ 3–3.1 and p*K*_a2_ ∼ 7.7–8.3, and sulfate p*K*_a_ ∼ 2.5).^43–46^

The morphology of NFC gels was first studied by using scanning electron microscopy (SEM). To preserve the microstructure of the gels, critical point drying was employed in sample preparation.^47^ In all compositions, the gel network consisted of fibrils that were most frequently between 10 and 30 nm in diameter and had high aspect ratios (Figure 2). Nanosized pores were observed between the fibrils. These results are similar to what has previously been reported for wood-derived NFCs.^48^

**Figure 2:**
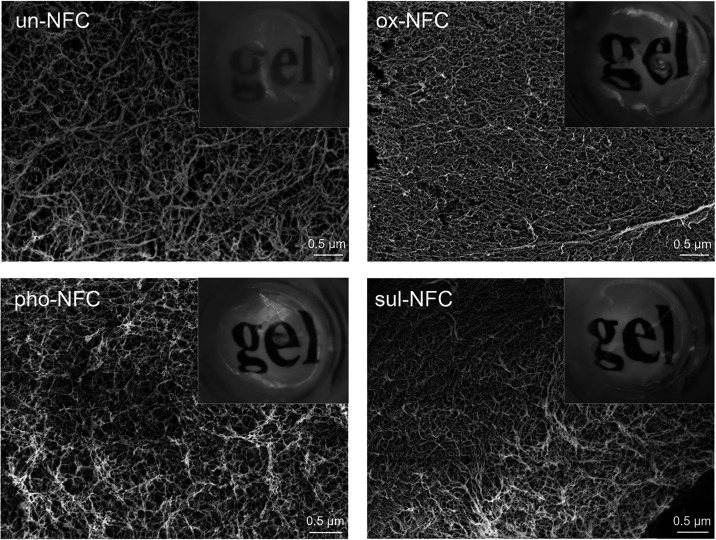
Microscopic and macroscopic appearance of NFC gels. SEM micrographs of critical-point-dried NFC gels showing the nanofibrillar architecture of NFC networks. The scale bar is 0.5 μm. The inset demonstrates the optical transparency of the NFC hydrogels.

Compared with un-NFC, the chemically modified NFC gels had a smaller average fibril diameter (16 nm vs 9–11 nm, Figure S1) and produced more transparent gels (Figure 2). This is likely due to the higher electrostatic repulsion between the negatively charged groups on the chemically modified NFC fibrils. Indeed, ζ-potential analysis confirmed the high negative surface charge of the chemically modified NFCs. All of the chemically modified NFC fibrils had ζ-potential values near −50 mV (Figure S2), indicating good colloidal stability. Fibrils of un-NFC also carried a negative charge, but it was considerably lower at −34 mV. The negative charge on unmodified NFC fibrils is not an uncommon finding, and has been attributed to the presence of residual hemicelluloses from pulping.^49^

### 3.2 NFCs Form Gels with Adjustable Mechanical Properties

As the effect of material mechanical properties on immune cell phenotype regulation is well recognized,^50^ we evaluated the mechanical properties of the differently functionalized hydrogels using oscillatory shear rheological measurements. Here, we defined stiffness as the average shear storage modulus (*G*′, the elastic component). The NFC gels used in this study originally had solid contents between 0.5 and 1.5%, leading to gels with varying degrees of stiffness (Table S1). To investigate the impact of NFC surface modification while minimizing the influence of stiffness on cell behavior, we adjusted the NFC concentrations so that all gels fell within a comparable stiffness range. As common 3D culture matrices such as collagen, Matrigel, and fibrin are often used at concentrations with *G′* around 0.1–0.2 kPa, we aimed for the NFC gels to have a similar stiffness.^51–53^ After concentration adjustment with water, *G′* of all gels was between 0.12 and 0.17 kPa (Table S1). This stiffness range was optimal to enable pipetting while simultaneously providing sufficient mechanical support for 3D cell culture.

Moreover, *G*′ was largely independent of the frequency, indicating the formation of strong networks in all gels (Figure 3a). This notion was supported by the observation that *G′* was over a magnitude higher than the shear loss modulus (*G″*, the viscous component), which was reflected in low phase angle values between 4.9 and 7.9 degrees in stiffness-adjusted NFC gels (Table S1). A low phase angle indicated the predominantly solidlike nature of the gels. Despite having a slightly lower *G′* than the other gels, pho-NFC had the lowest phase angle. In contrast, the phase angle was higher in un-NFC compared to the chemically modified gels, which might be due to the presence of a discontinuous floc network in the unmodified NFC gel.^54^ Furthermore, chemically modified NFCs formed gels of similar strength at lower solid contents than un-NFC. This is likely due to the increased entanglement of the fine fibrils in chemically modified NFC gels, resulting in stronger networks.^55^ Additionally, all gels had high viscosity at rest but showed a strong shear-thinning behavior upon increasing strain (Figure 3b), a characteristic feature of NFC gels and suspensions.^56^ The onset of shear thinning occurred at slightly lower strains in un-NFC and pho-NFC than in ox-NFC and sul-NFC, suggesting differences in internal structuring of the gels in response to strain.^57^ Thus, while the rheological analysis did reveal minor differences between the gels, the overall mechanical responses were comparable, and the stiffness-dependent effects were effectively minimized for cell culture studies.

**Figure 3:**
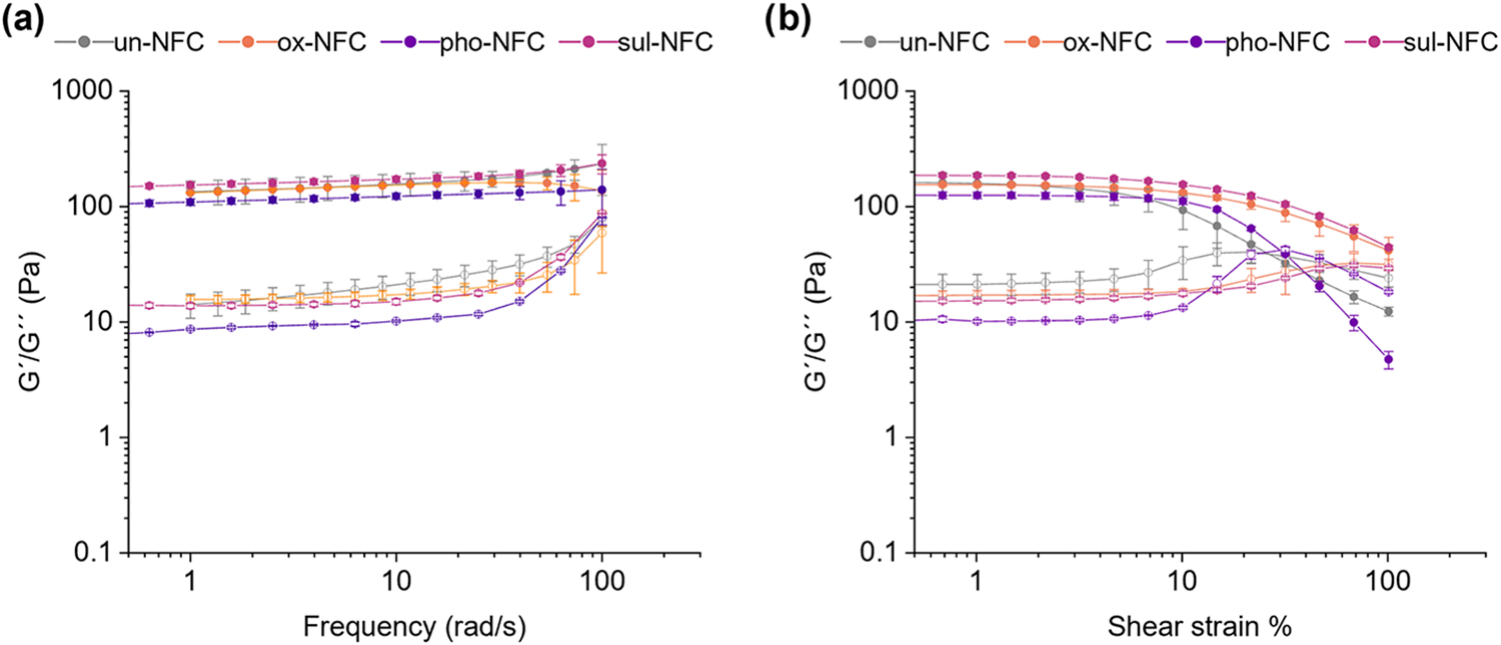
Rheological behavior of NFC gels after stiffness adjustment by controlling the concentration. (a) Frequency sweep at 1% strain and (b) strain sweep at 10 rad/s. The measurements were performed at 37 °C. The shear storage modulus *G′* is represented by filled circles and the shear loss modulus *G″* is represented by open circles. The points indicate average values with standard deviation as error bars (*n* = 3). Due to the low standard deviation, the error bars for some points may not be clearly distinguishable. NFC gel concentrations were adjusted to be in a similar stiffness range before the measurement, using 1.125% un-NFC, 1% ox-NFC, 0.5% pho-NFC, and 0.75% sul-NFC.

### 3.3 NFC Gels Have Varying Effects on Immune Cell Phenotypes

For our initial biological studies, we explored whether the surface chemistry of NFC gels regulated the phenotype of human PBMCs in 3D culture. PBMCs consist of a mixture of naïve immune cells, including monocytes, NK cells, T cells, and B cells. To evaluate whether the NFC gels could steer their behavior, we used a commercial NanoString panel to profile the expression of 770 genes involved in immune cell regulation. The cells showed distinct gene expression profiles after just 2 days of culture in the different NFC gels (Figure 4a). However, since PBMCs from only one donor were used for the preliminary screening, it was not possible to reliably determine differentially expressed genes betweenthe different conditions. Instead, we compared the expression of genes that are commonly used to distinguish M1 from M2 macrophages to gain insights into potential macrophage polarization within the NFC gels. M1 markers *CD80* and *CD86* were detected at similar levels in NFC gels as in the gel-free control (Figure 4b), indicating that in the absence of proinflammatory cues like lipopolysaccharide (LPS), M1 macrophage polarization was negligible. In contrast, M2 markers *CD163* and *MRC1* (encoding mannose receptor CD206) showed more variable expression between the culture conditions (Figure 4c). This suggested that the surface chemistry of NFCs may indeed differentially affect monocyte conversion toward M2-like macrophages.

**Figure 4:**
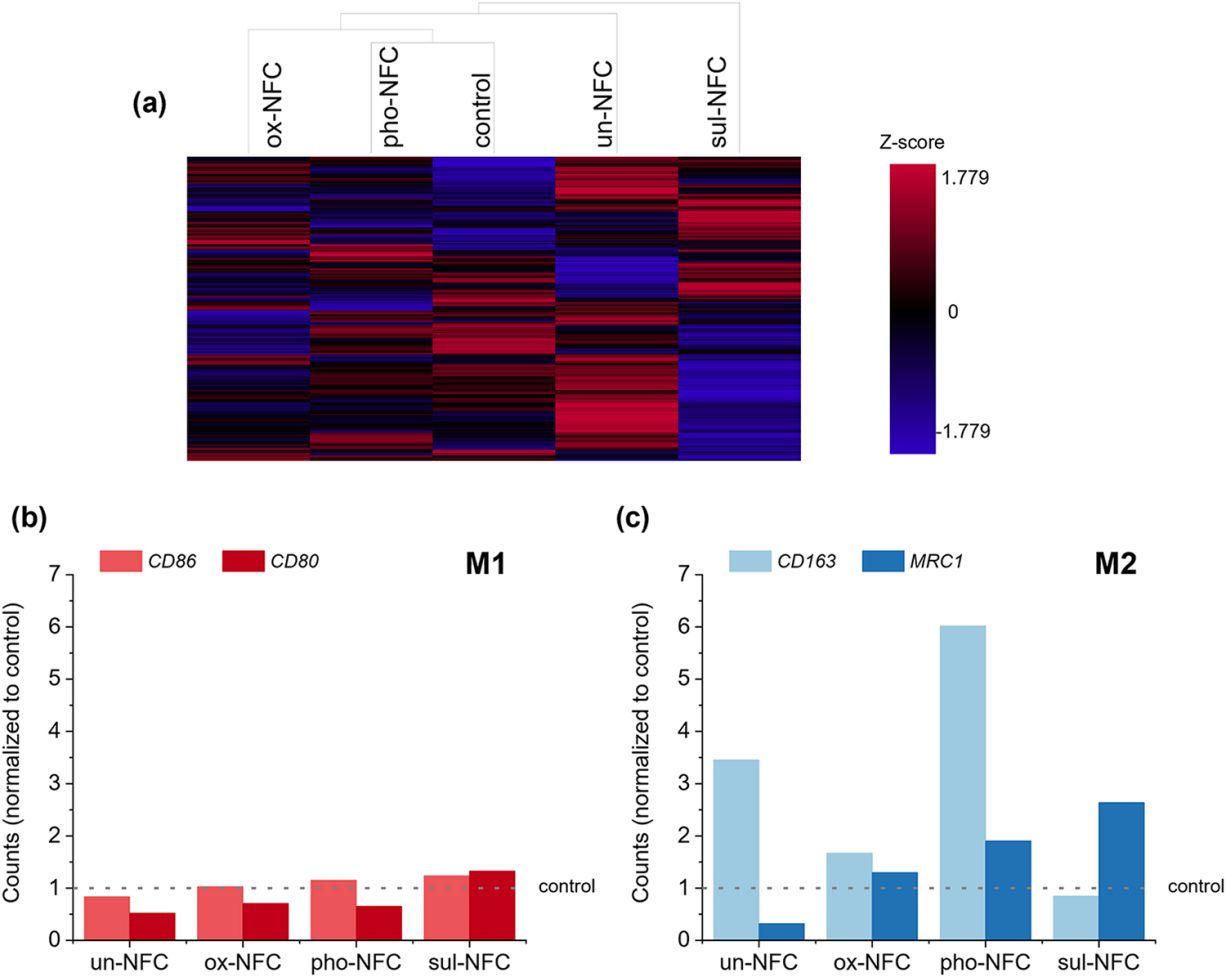
Effect of NFC gels on PBMCs’ gene expression. (a) Agglomerative cluster heatmap showing different gene expression profiles for PBMCs cultured in ultralow attachment plates in suspension (control) or in 3D NFC gels for 2 days. (b) Expression of genes associated with M1 macrophages (*CD80*, *CD86*) and (c) M2 macrophages (*CD163*, *MRC1*) in PBMCs. The counts were normalized to the counts of the respective genes in the control. Gene expression profiling was performed using a NanoString panel (*n* = 1 donor).

As PBMCs contain only ∼10–30% monocytes, we continued with the development of 3D NFC culture model based on monocyte-only cultures to isolate the specific effects of NFCs on macrophage polarization. The monocytes were isolated from the PBMCs using positive selection with anti-CD14 conjugated magnetic microbeads, which results in high-quality monocyte populations.^58^ As a control, cells were cultured on conventional 2D tissue culture plates. No extra growth factors or cytokines were supplemented into the cell culture medium in any of these conditions.

First, we evaluated the effect of 3D culture in NFC gels on monocyte viability. Because nanomaterials can interfere with cell health assays,^59^ we utilized a combination of three different readouts to study cell survival within 24 h of seeding. Cytotoxicity was assessed using the CellTox assay, which utilizes a fluorescent, membrane-impermeable DNA-binding dye that is excluded from live cells and thus stains only dead cells with compromised membranes regardless of cell death mode (Figure 5a). Cell death was comparable in un-NFC, ox-NFC, and pho-NFC (27–40% of cytotoxicity control) and 2D culture (27% of cytotoxicity control), indicating that the NFCs were not cytotoxic to monocytes. Cells in sul-NFC displayed more variability in cell death, mainly because cells from one donor did not respond well to culture in this condition. In addition, cell viability was evaluated by the metabolic activity of living cells using CellTiter Glo assay (ATP production) and AlamarBlue (reduction ability) (Figure 5b). The average ATP content was 1.5–3 times lower in cells in 3D NFC gels than in 2D cultured cells. However, cells in chemically modified 3D NFC gels had a similar reduction ability of AlamarBlue as 2D cultured cells (>85% of 2D control). In contrast, cells in un-NFC reduced only 50% of AlamarBlue compared to 2D cultured cells. The differences in the viability results from the ATP-based assay and AlamarBlue assay might be due to alterations in the metabolic activity of the cells, which can occur during monocyte activation and macrophage differentiation.^60^ Altogether, the CellTox cytotoxicity assay and viability assays indicated that while some monocytes were lost early in the culture, monocytes could survive in 3D NFC gels and showed different metabolic activities depending on NFC surface modification.

**Figure 5:**
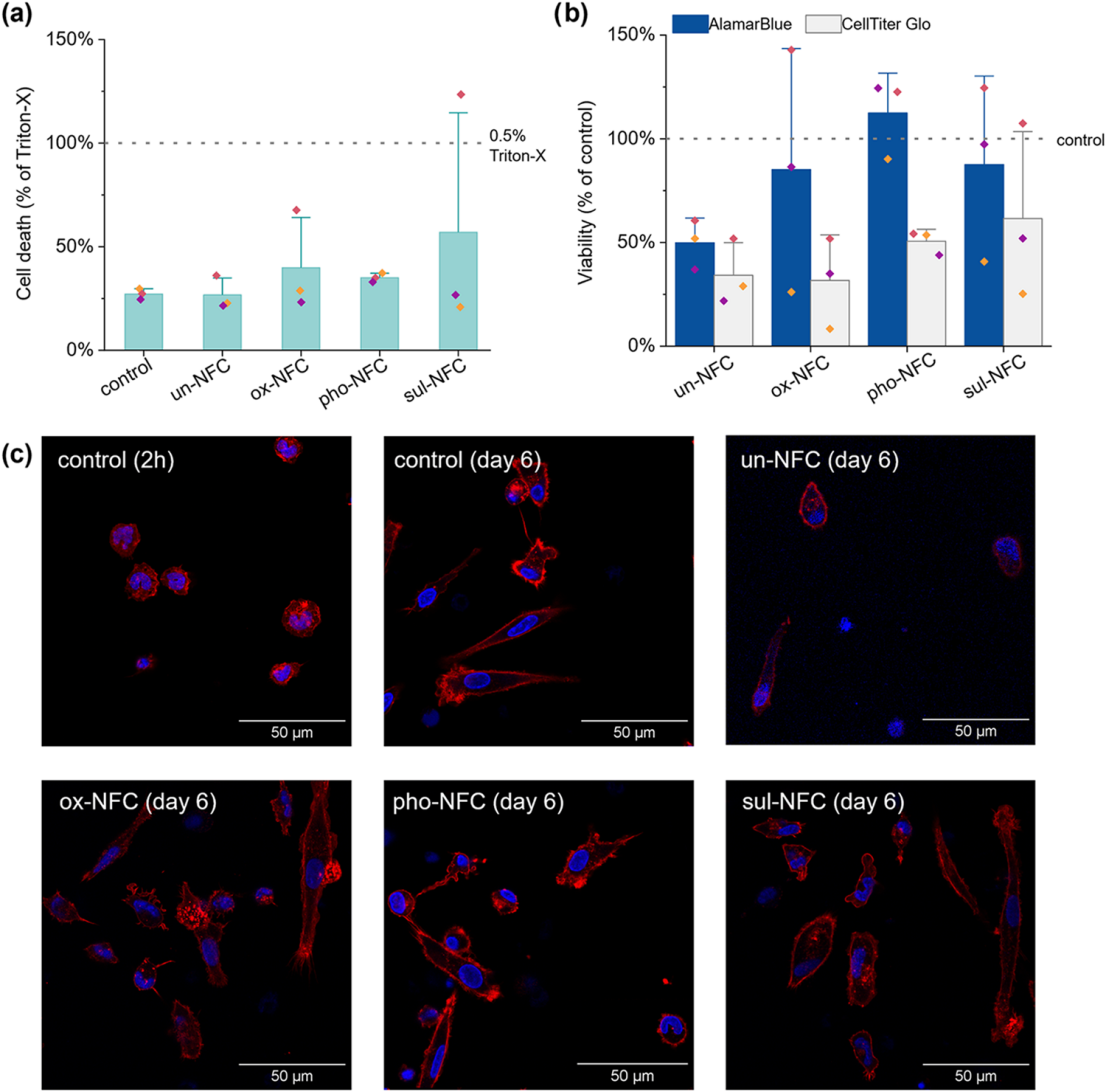
Effect of NFC gels on monocyte viability. (a) Column chart of cytotoxicity (CellTox assay) and (b) metabolic activity of viable cells (AlamarBlue: blue columns and CellTiter Glo: white columns) of monocyte-derived cells cultured for 24 h on a 2D tissue culture plate (control) or in 3D NFC gels. Cell death was normalized to the cytotoxicity control where cells had been lysed (0.5% Triton X-100), and metabolic activity was normalized to 2D cultured cells. The columns indicate average values with standard deviation as error bars, with individual donors represented by different colored dots (*n* = 3 donors). (c) Immunofluorescence confocal microscopy of monocyte-derived cells after 2 h or 6 days of culture. F-actin staining is shown in red, and nuclei in blue (*n* = 3 donors). The scale bar is 50 μm.

Next, we prolonged the monocyte culture period to 6 days, which is a common duration for differentiating monocytes into macrophages. Since monocyte differentiation is typically associated with changes in cell morphology,^61^ we compared the cell appearance in the beginning to the appearance at the end of the culture period. The chemically modified NFC gels were transparent and were thus more suited for light microscopy than the opaque un-NFC gel (Figure S3). On day 0, monocytes were round and homogeneous in size (Figures 5c and S3). After 6 days, the density of cells in the un-NFC gel seemed lower than in chemically modified NFC gels or in 2D culture, correlating with the viability results where a decrease in ATP was not matched with the maintenance of cellular reduction ability. In all conditions, the cells displayed notable morphological heterogeneity, with round cells mixed with spread and highly elongated cells (Figure 5c), suggesting monocyte differentiation.

### 3.4 Negatively Charged NFC Gels Promote Generation of M2-like Macrophages

Next, we investigated whether the monocyte-derived cells cultured in the different 3D NFC gels expressed M2 macrophage-associated cell surface receptors at the protein level. After 6 days in culture, NFC gels were softened with a brief cellulase treatment to release cells from the matrix, and the cells were subsequently stained for M2 markers CD163 and CD206 and analyzed with flow cytometry. We also assessed the expression of CD14, which is a monocyte marker whose levels change during macrophage differentiation.^62^ As a control, 2D cultured cells detached from the tissue culture plate were stained to determine the baseline of monocyte-macrophage conversion in the absence of any gel matrix. Debris, cell doublets, and dead cells were excluded prior to analysis (Figure S4).

The flow cytometry analysis revealed variation in the expression of macrophage surface markers across donors (Figure 6a–d), which may reflect differences in donor characteristics and, thus, influence cell responses to the culture conditions. Despite these variations, noticeable trends were observed between cells cultured in 2D compared to the cells in different 3D NFC gels. On average, the percentage of cells expressing CD14 was higher in 2D cultured cells compared to cells in most of the NFC gels (Figure 6a). The exception was pho-NFC, which had a higher percentage of CD14+ cells than the 2D control. According to the Kruskal–Wallis test with Dunn’s pairwise comparison, pho-NFC had a significantly higher proportion of CD14+ cells compared to un-NFC and sul-NFC (Bonferroni-adjusted *p*-value = 0.023). In all conditions, the average percentage of CD14+ cells was below 50%, suggesting that some of the cells were differentiating away from classical monocytes.^63^ Regardless, the median fluorescence intensity (MFI) of the CD14+ population was comparable in all conditions (Figure 6b). The changes in the MFI of CD14 were relatively modest, with most values being 0.5–1.5-fold of the 2D control.

**Figure 6:**
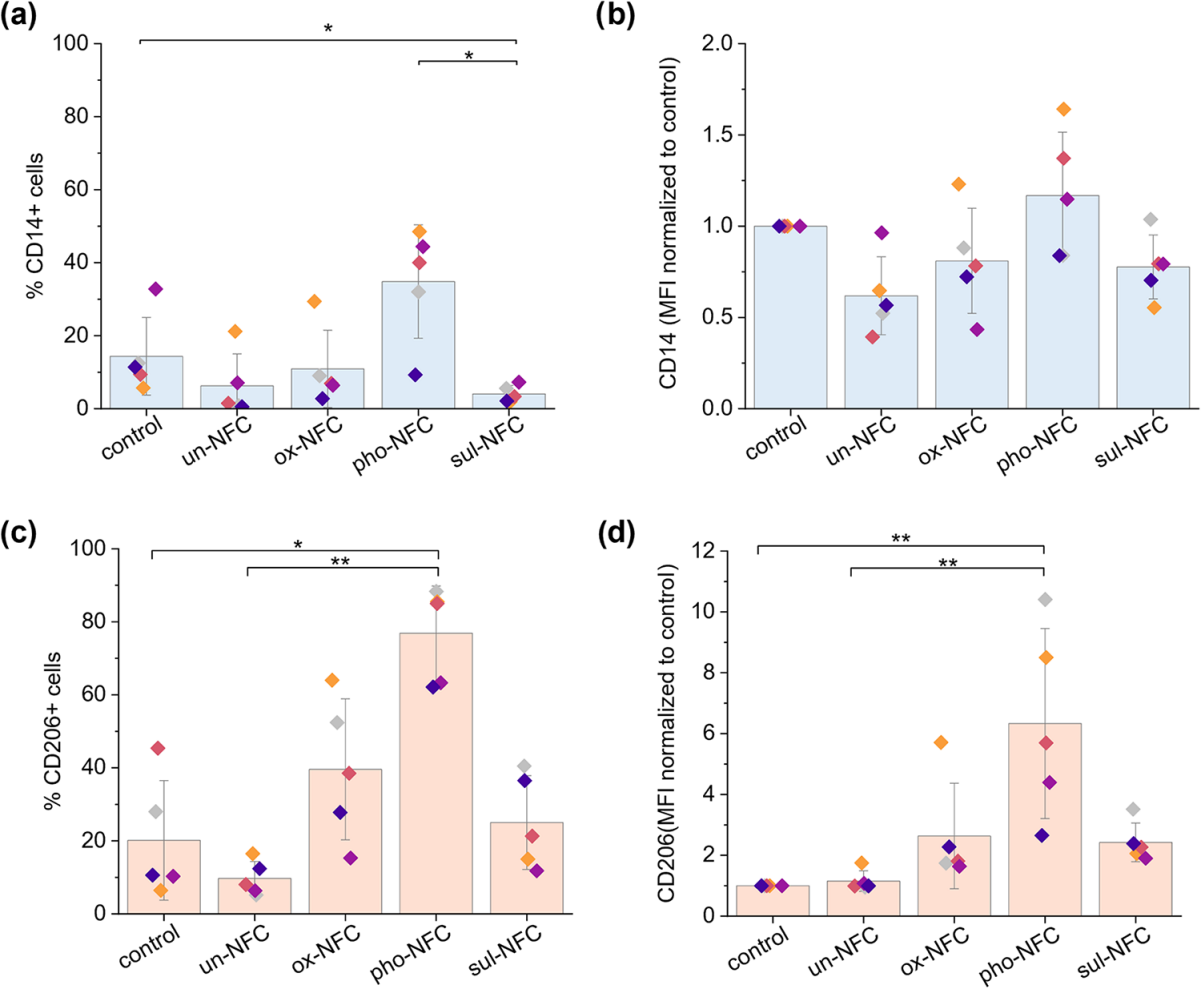
Flow cytometry analysis of CD14 and CD206 expression in monocyte-derived cells cultured for 6 days on a 2D tissue culture plate or in 3D NFC gels. (a) Percentage of CD14+ cells in different conditions. (b) Median fluorescence intensity (MFI) in the CD14+ population. (c) Percentage of CD206+ cells in different conditions. (d) Median fluorescence intensity (MFI) in the CD206+ population. The MFI values in each condition were normalized to the MFI of the respective donor in 2D culture (control). The columns indicate average values with standard deviation as error bars, with individual donors represented by different colored dots (*n* = cells from 5 donors). Asterisks denote groups with significant differences between each other in the Kruskal–Wallis test, with *p < 0.05 and **p < 0.01 after Bonferroni correction.

The expression of the M2 marker CD206 was more strongly affected by the culture conditions than CD14 (Figures 6c,d and S5). While the proportion of CD206+ cells and the MFI in CD206+ population cells were similar in the 2D control and in the un-NFC gel, cells in chemically modified NFC gels showed an increase in these values. Importantly, the extent of CD206 upregulation in the chemically modified NFC gels was greatly influenced by the specific surface modification of NFC. The induction efficiency of CD206 followed the order pho-NFC ≫ ox-NFC > sul-NFC. The percentage of CD206+ cells in pho-NFC was on average ∼80%, followed by ∼40% in ox-NFC and ∼25% in sul-NFC, with the 2D control having only ∼20% CD206+ cells. Correspondingly, the average MFI of CD206 increased 2.6-fold in ox-NFC and 2.4-fold in sul-NFC compared to the 2D control. For cells in pho-NFC, the average upregulation was as high as 6.3-fold compared to the 2D control. Furthermore, the difference in CD206 expression was significant between cells in pho-NFC compared to the 2D control (Bonferroni-adjusted *p-*value = 0.002) and un-NFC (Bonferroni-adjusted *p*-value = 0.007). Expression of CD14 and CD206 was also verified with immunofluorescence confocal microscopy (Figure S7). This is also in line with the results from NanoString gene expression analysis with PBMCs. Although *CD163* gene expression was also upregulated in PBMCs, no considerable CD163 protein expression was observed in any of the conditions with monocyte-derived cells (Figure S5), which may be due to differences in translational regulation or the culture setup.

The CD206+ CD163– phenotype resembled M2a macrophages, which are commonly obtained after culturing monocytes in the presence of the macrophage colony-stimulating factor (M-CSF) and IL-4.^8^ Therefore, we stimulated the cells in 2D culture with these factors to compare the phenotype of M2a macrophages with the phenotype observed in pho-NFC, which showed the most efficient induction of CD206 among the studied NFC gels. For this comparison, the cultures in 2D and pho-NFC were repeated with additional donors, explaining the variation in pho-NFC’s efficiency to upregulate CD206 expression (6.3-fold and 2.3-fold compared to the 2D control, as shown in Figures 6d and 7b, respectively). The IL-4-differentiated macrophages showed no CD163 expression but had lower CD14 and higher CD206 expression than cells cultured in 2D without any added compounds (Figure S7). Remarkably, cultures in the pho-NFC gel had comparable CD206 expression levels with IL-4-supplemented 2D cultures (Figure 7a,b**)**. This strongly indicated the generation of M2-like macrophages in pho-NFC. In addition, CD206 induction in this gel was more consistent than with IL-4 stimulation, evidenced by the higher average percentage of CD206+ cells and the relative MFI (Figure 7a,b).

**Figure 7:**
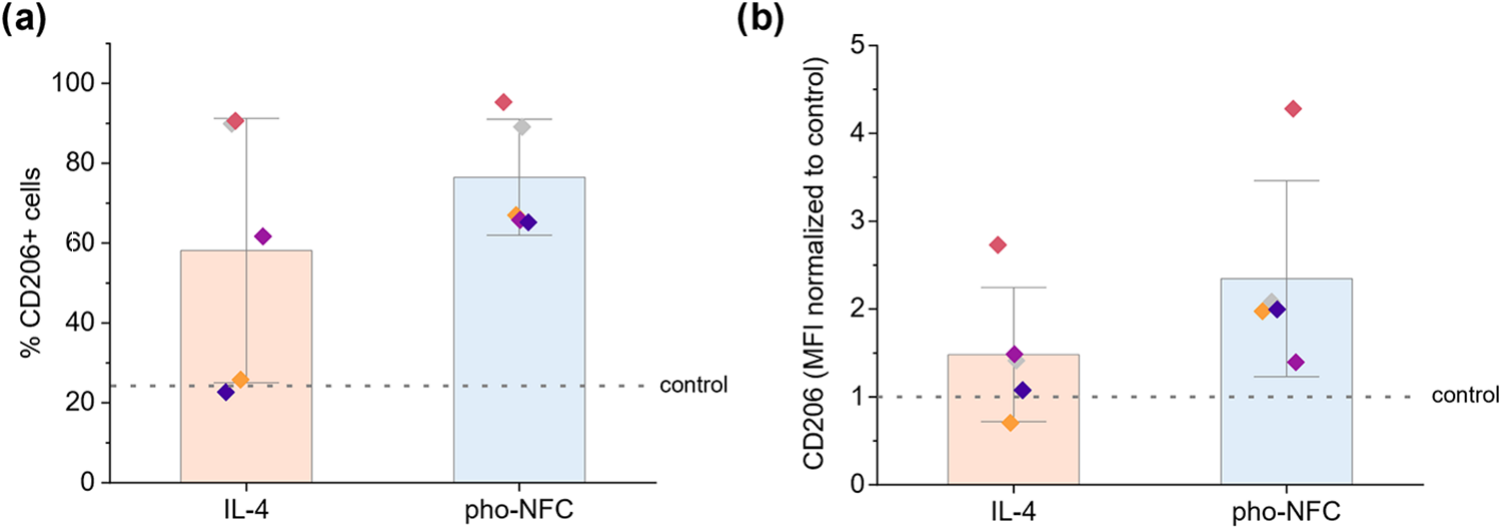
Flow cytometry analysis of CD206 expression in the 3D pho-NFC gel compared to IL-4-stimulated 2D cultured cells. Monocytes were cultured for 6 days in 3D pho-NFC or differentiated into M2a macrophages by culturing monocytes on a 2D tissue culture plate in the presence of M-CSF and treating the cells with IL-4 on day 3. (a) Percentage of CD206+ cells in each condition. (b) Median fluorescence intensity (MFI) in the CD206+ population. The MFI values in each condition were normalized to the MFI of the respective donor in 2D culture without stimulation (dotted line, control). The columns indicate average values with standard deviation as error bars, with individual donors represented by different colored dots (*n* = cells from 5 donors). The differences between the two conditions were not statistically significant in the Kruskal–Wallis test.

### 3.5 Phosphate-Modified NFC Gel Modulates Macrophage Cytokine Secretion

Intrigued by the ability of pho-NFC to generate CD206+ CD163– macrophages in the absence of stimulation by IL-4 or other cytokines, we selected this NFC culture system for further investigations. To assess the functional differences between cells in pho-NFC and in 2D culture, we evaluated the cytokine secretion profiles using enzyme-linked immunosorbent assay (ELISA). Cytokines associated with both anti-inflammatory and proinflammatory responses were quantified, as M2-like macrophages are phenotypically heterogeneous and may secrete different types of cytokines depending on the context.^64^ As an additional comparison, 2D cultured cells were stimulated with polarization factors to induce either anti-inflammatory or proinflammatory responses. At the end of the culture, the anti-inflammatory IL-10 and transforming growth factor β 1 (TGF-β1) and the proinflammatory tumor necrosis factor α (TNF-α) and IL-1β were measured from the cell culture medium supernatant.

The potential entrapment of the cytokines into the NFC matrix was first investigated by incubating cell-free pho-NFC with cytokine solutions and measuring their concentration in the supernatant.^65^ The concentrations of IL-1β, IL-10, and TGF- β1 were similar irrespective of the presence or absence of the gel, indicating that cytokine entrapment into pho-NFC did not occur and confirming that ELISA results were comparable across 2D and 3D cultures. For TNF-α, a slight retention in pho-NFC was observed (approximately 30%).

To set the baseline for anti-inflammatory cytokine secretion, IL-4 stimulation was used to promote the M2a macrophage phenotype in 2D culture. While TGF-β1 was below the detection limit of the ELISA kit (31.2 pg/mL) in all conditions, cells in both unstimulated and IL-4-stimulated 2D controls secreted IL-10 at comparable levels (682 and 649 pg/mL) (Figure 8a). However, cells in pho-NFC secreted on average 799 pg/mL IL-10, which was 1.2-fold higher than in the 2D control.

**Figure 8:**
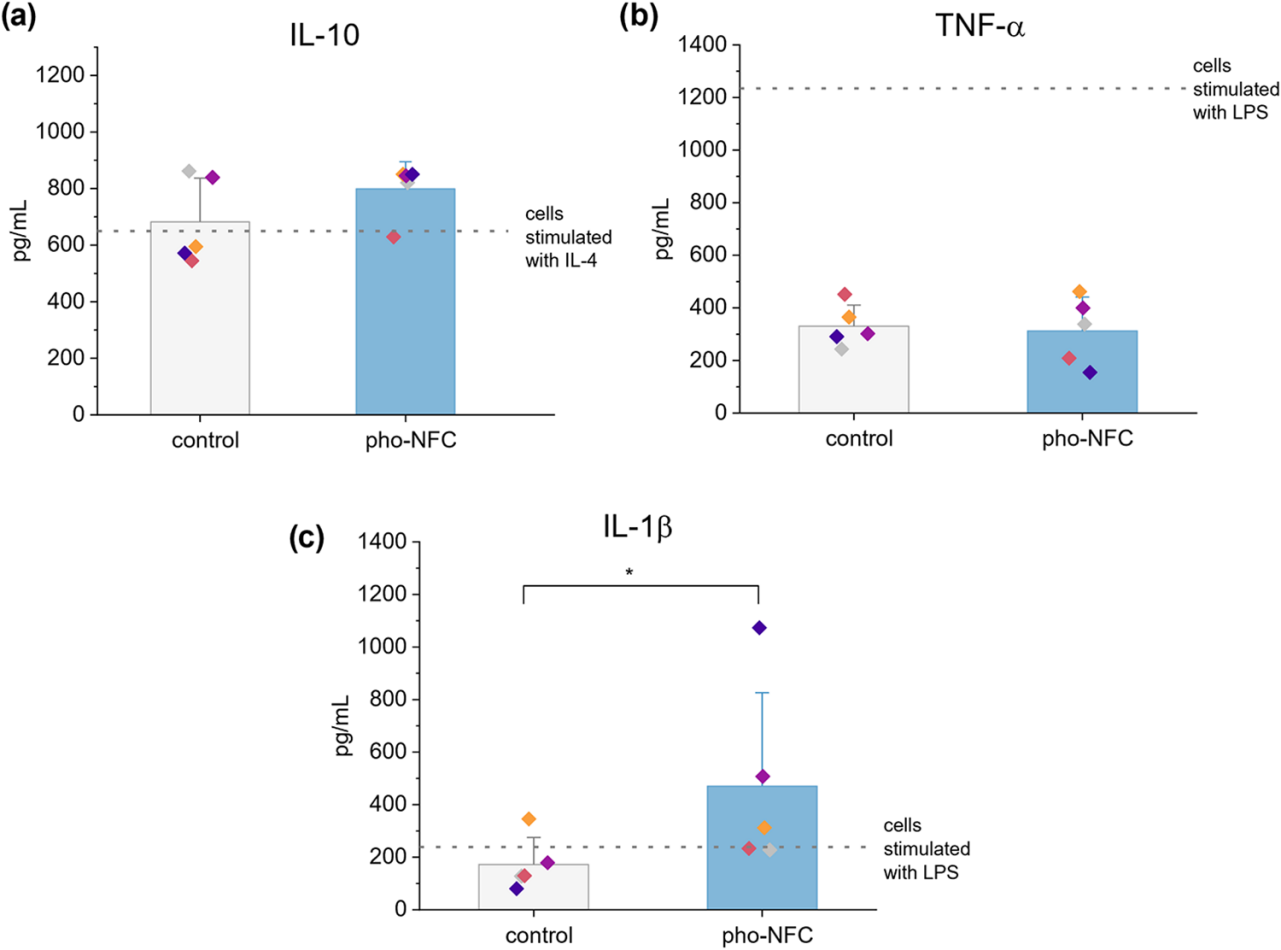
Cytokine secretion of monocyte-derived cells. Monocytes were cultured for 6 days on a 2D tissue culture plate (control) or in 3D in pho-NFC gels, after which (a) IL-10, (b) TNF-α, and (c) IL-1β in the supernatant were quantified with ELISA. The columns indicate average values with standard deviation as error bars, with individual donors represented by different colored dots (*n* = 9 donors, 5 per cytokine). The Kruskal–Wallis test was performed to compare cytokine levels in the unstimulated 2D control and pho-NFC. Asterisks denote groups with significant differences, with *p < 0.05. For comparison, cells were also cultured with M-CSF and stimulated with LPS (for proinflammatory cytokines, *n* = 3) or IL-4 (for anti-inflammatory cytokines, *n* = 5). The dotted line marks the average cytokine concentration in the stimulated 2D cultured cells.

The secretion levels of proinflammatory cytokines in 3D pho-NFC were compared to those of 2D cultured cells stimulated with proinflammatory LPS. TNF-α secretion levels in both unstimulated 2D cultured cells and cells in pho-NFC were approximately 4-fold lower than in LPS-stimulated cells, with average concentrations of 331 and 312 pg/mL, respectively (Figure 8b). Even when accounting for TNF-α retention in the NFC matrix, the differences between 2D cultured and pho-NFC cultured cells were not statistically significant. In contrast, significant differences in IL-1β secretion were observed between unstimulated 2D cultured cells and pho-NFC cultured cells, resulting in 2.6-fold induction in pho-NFC (172 vs 471 pg/mL, *p* = 0.047). LPS stimulation did not lead to significant increases in the secretion of IL-1β (Figure 8c). Altogether, the ELISA results show that 3D culture in pho-NFC promotes macrophage phenotype having secretory activity that is distinct from that of 2D cultured cells with or without stimulation.

### 3.6 Phosphate-Modified NFC Gel Supports the M2-like Macrophage Phenotype Independent of Protein Adsorption

Next, we looked in more detail into the possible explanation behind the ability of pho-NFC to modulate the macrophage phenotype. It is widely recognized that chemical modifications can alter protein adsorption onto nanomaterials, which then affects cellular responses, like adhesion and differentiation.^66^ As NFC gels are composed of high-aspect-ratio fibrils, they provide a high surface area for the adsorption processes. To evaluate the presence of such interactions, we measured the amount of adsorbed protein and the surface charge of NFC gels after incubating them with various cell culture relevant solutions. When incubated with the complete cell culture medium (Mammocult), all NFC gels retained similar amounts of protein, as determined by colorimetric protein assay of the supernatant (Figure 9a). Adsorption of proteins was also supported by the ζ-potential measurements, where a higher increase in positive charge was observed in NFCs incubated with Mammocult compared to when NFCs were measured in water (Figure 9b). The positive charge increased less when the ζ-potential was measured in PBS, suggesting that the presence of ions alone was not responsible for screening electrostatic repulsions between NFC fibrils.

**Figure 9:**
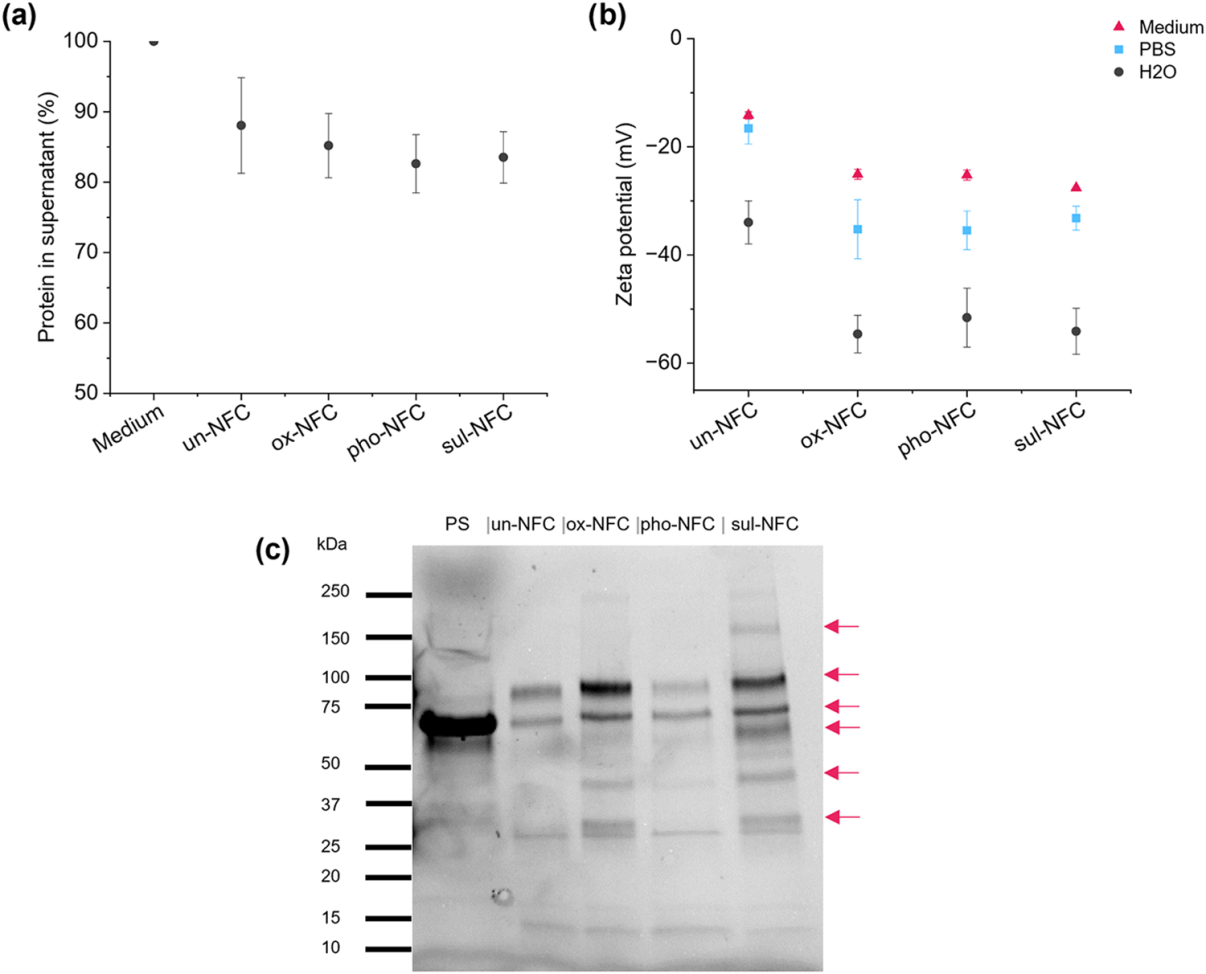
Protein interactions with NFCs. (a) Protein concentration in the supernatant after incubating 0.5% NFCs with the Mammocult medium for 30 min at 37 °C. The protein concentration was measured with Lowry assay, and the values were normalized to the protein content in the Mammocult medium. The points indicate average values with standard deviation as error bars (*n* = 3). (b) ζ-potential of 0.01% NFCs measured in water, 0.1× PBS, or 0.1× Mammocult medium. The points indicate average values with standard deviation as error bars (*n* = 3). (c) Representative SDS-PAGE of 0.5% NFCs incubated with PS (*n* = 3). Red arrows indicate positions of protein bands that showed differences among the NFCs. PS = protein supplement.

To obtain qualitative insights into the composition of the adsorbed protein layer on NFC fibrils (protein corona), the gels were incubated with Mammocult, after which weakly adsorbed proteins were removed with repeated washes. The remaining proteins were desorbed from NFCs and separated by SDS-PAGE. Since Mammocult contained 10% serum-free protein supplement (PS), the protein concentration was too low to be visualized in SDS-PAGE (Figure S8a). Therefore, NFC gels were incubated with 100% PS to increase the amount of detectable protein. For comparison, we also incubated the NFC gels with FBS, which is a common supplement in various cell culture media. With FBS, the protein corona on chemically modified NFCs was very diverse and un-NFC behaved as an antifouling material (Figure S8b). With PS, the differences between NFCs were smaller but still noticeable (Figure 9c). For example, all chemically modified NFCs had a band at 40 kDa, which was missing from un-NFC. Among the chemically modified NFCs, pho-NFC showed the lowest protein intensity and sul-NFC the highest. Compared to the PS control, which showed a prominent band at 50–70 kDa, likely corresponding to albumin, the NFCs showed two bands at the same region. While in un-NFC, ox-NFC, and sul-NFC, the optical density of the upper band was similar or higher than that of the lower band, pho-NFC showed an opposite pattern (Table S2). Thus, although SDS-PAGE analysis did not reveal large differences in protein adsorption when defined serum-free PS was used, some variations in the protein corona were observed depending on the chemical modification of NFC.

Next, we investigated whether the slightly different protein corona on pho-NFC was responsible for converting monocytes into M2-like macrophages. For this experiment, monocytes were cultured in pho-NFC using either complete culture medium containing 10% PS or reduced protein medium with only 1% PS. The expression of CD206 was comparable in both protein concentrations (Figure 10a). While it is possible that cell-secreted proteins may be involved in NFC-cell interactions, this result suggests that cell culture medium proteins were not the main drivers of M2-like macrophage generation.

**Figure 10:**
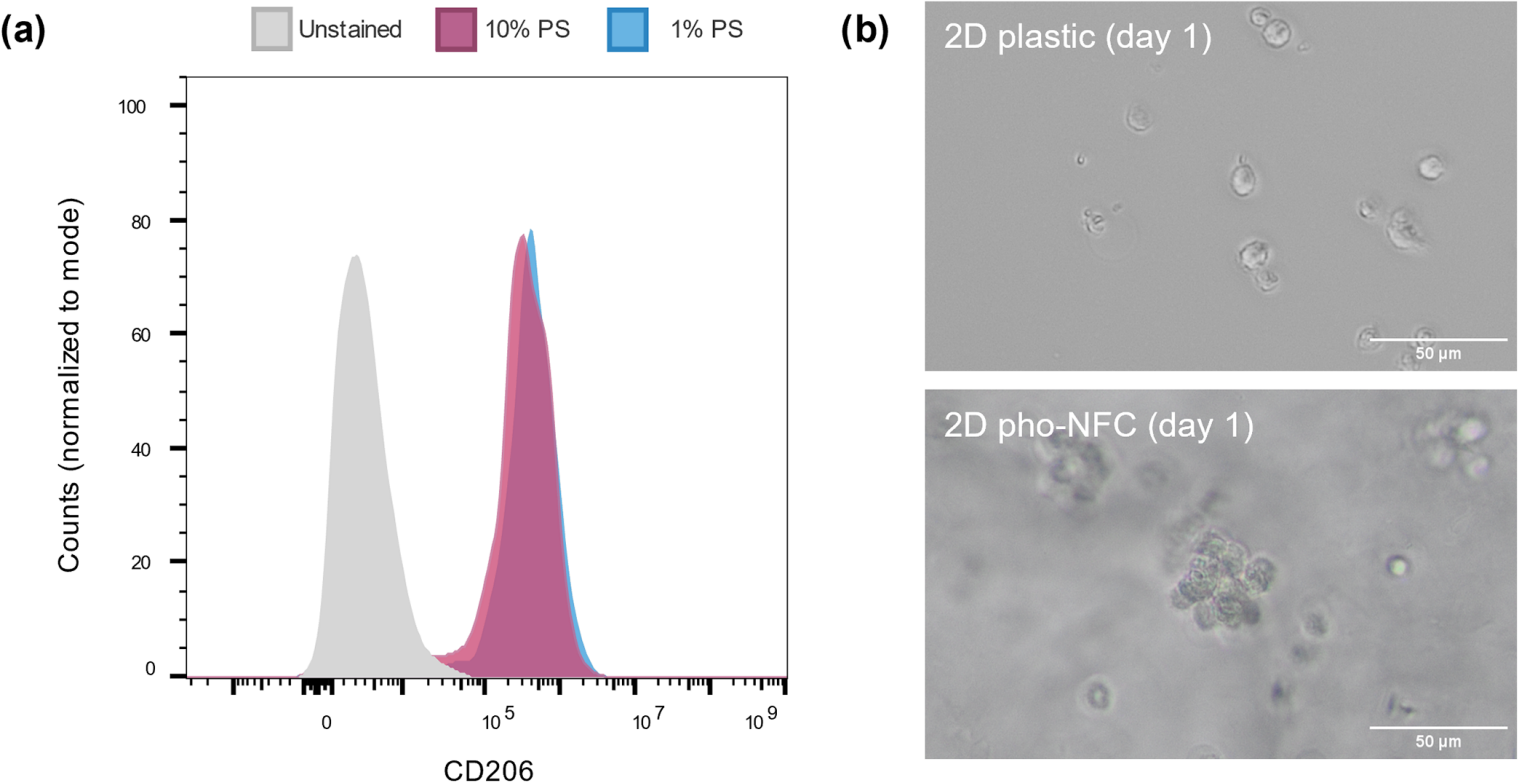
Contribution of proteins and cell adhesion to the regulation of the cell phenotype by pho-NFC. (a) Representative flow cytometry histograms of CD206 expression of monocytes cultured for 6 days in 3D pho-NFC in Mammocult containing either 10% or 1% proliferation supplement (PS). The red histogram represents stained cells cultured in the standard medium, the blue histogram represents cells cultured in the protein-reduced medium, and the gray histogram represents the unstained cells (*n* = 5). (b) Representative light microscopy images of cell morphology after 24 h culture on 2D pho-NFC or on a plastic slide (*n* = 3). A standard medium with 10% PS was used. The scale bar is 50 μm.

Finally, we studied the possible role of adhesion in mediating the immunomodulatory effects of pho-NFC. Adhesion to pho-NFC was evaluated by culturing monocytes on top of the 2D gel layer instead of embedding the cells inside the gel for 3D culture. After 24 h, the cells on 2D pho-NFC had formed clusters, whereas monocytes cultured on the 2D plastic substrate remained as single cells (Figure 10b). The formation of clusters indicates that the cells adhered weakly to pho-NFC. Consequently, the clusters easily detached from pho-NFC during fixation, preventing their staining for immunofluorescence microscopy. However, clusters with similar morphology were observed in the 3D pho-NFC gel, and the cells in these clusters stained positive for CD206 (Figure S9). Collectively, the results suggest that the promotion of M2-like macrophages in pho-NFC gels is a direct effect of surface chemistry rather than an indirect effect mediated by protein adsorption or cell adhesion.

## 4 Discussion

Regulation of macrophage function is an important consideration in various areas of biomedicine, including tissue regeneration and the development of 3D culture models for *ex vivo* investigation of innate immunity in cancer research and immunological diseases. Discovery of hydrogel materials that allow the 3D cell culture of human monocytes/macrophages is expected to advance our understanding of microenvironment-guided macrophage polarization. This, in turn, could lead to the development of more accurate immunological models compared with conventional 2D cultures. However, 3D immune cell culture models are most often based on animal-derived matrices,^21,67,68^ highlighting the need for better-defined, scalable, and cost-efficient hydrogel materials to study macrophage phenotypes *in vitro*.

The current study explored the potential of plant-derived NFC gels with varying surface modifications to generate material-guided macrophages within a 3D environment. Although NFC gels are not components of native tissue, they are known to recapitulate the 3D fibrillar organization and the viscoelastic nature of the ECM. These features are important regulators of the cell phenotype but are absent in 2D monolayer culture. Moreover, it has been established that changing the functional group on polysaccharides and other materials alters their bioactivity.^33,69–71^ Yet, a gap of knowledge remains on whether NFC fibrils and their surface modifications can modulate monocyte-macrophage conversion in 3D culture. Given the importance of anti-inflammatory macrophages in cancer progression and tissue regeneration, our focus was primarily on generating M2-like macrophages within the NFC gels. For this, primary human monocytes from peripheral blood were cultured in ox-NFC, pho-NFC, and sul-NFC, and the viability and phenotype of the cells were compared to cells cultured in the un-NFC gel and 2D culture. The conversion of monocytes into M2-like macrophages was evaluated mainly by the expression levels of the mannose receptor CD206 whose upregulation is associated with the anti-inflammatory macrophage phenotype.^2^ All chemically modified NFC gels induced higher CD206 expression than the 2D cell culture, demonstrating the importance of placing the cells within the context of a 3D environment. Notably, the different chemical modifications on the nanofibril surface further stimulated macrophage phenotype conversion of macrophages. Among all conditions, the pho-NFC gel was most efficient in inducing CD206 expression. On the contrary, the monocyte cultures in the un-NFC gel led to poorer viability and low CD206 expression.

Since one of the key differences between chemically modified and unmodified NFC gels was the surface charge density, the improved performance of chemically modified NFC gels might be related to the higher negative charge on the fibril surface. Surface charge is recognized as an important factor influencing cell interactions with biomaterials.^72^ For example, tissue culture-treated plastic used to promote cell attachment also exhibits negative charge due to the plasma treatment of polystyrene.^73^ In line with this, several studies have demonstrated that various types of mouse and human cells show lower adhesion to unmodified NFC substrates than to anionic NFC substrates with carboxylate, sulfate, or phosphate modifications.^74–77^ On the other hand, it has also been reported that THP-1 monocytes display similar adhesion to unmodified and carboxylate NFC substrates.^39^ The contradicting results may be related to differences in the cell-type-specific behavior or NFC material processing. In our system, primary monocytes did not appear to adhere well to pho-NFC, as the cells cultured on top of the gel formed multicellular clusters. As no cell fusion was evident, the clusters on pho-NFC did not resemble multinucleated giant cells that may form during foreign body reaction to implanted biomaterials *in vivo*.^78^

Since phosphorylation was more efficient than other modifications in promoting CD206 expression, it is clear that the negative charge was not the only factor responsible for enhanced macrophage generation in the chemically modified NFC gels. Previously, it has been postulated that cells interact with materials indirectly through the proteins adsorbed from the cell culture medium.^66,79–81^ The composition and conformation of the adsorbed proteins may further be affected by the NFC surface modification.^80,82^ However, reduction of protein supplementation in the cell culture medium did not affect the ability of the pho-NFC gel to promote the generation of CD206+ macrophages. Similarly, the interaction of mesenchymal stem cells with sulfate- and carboxylate-modified NFC scaffolds has been shown to be unaffected by the serum in the medium.^75^ The ability to culture macrophages and other cells in plant-derived NFC gels using serum-free media is a great advantage for the development of xeno-free culture systems.

The stimulation of monocyte-macrophage conversion in the pho-NFC gel in a low-protein culture medium raises the possibility that surface-modified NFC might directly interact with cell surface receptors. Macrophages recognize various molecules from plants, microbes, and animals primarilythrough the pattern recognition receptors (PRRs), whose activation leads to changes in the inflammatory state of the cell.^83^ In biological systems, phosphate groups are part of various macromolecules, such as phospholipids and microbial and platelet-derived polyphosphates, which can steer macrophage phenotypes.^84–86^ In addition, phosphorylation can give anti-inflammatory properties to synthetic polymers, such as dendrimers, which are highly branched polymeric molecules with a modifiable surface. Poupot et al. showed that dendrimers terminating with phosphonic groups had a stronger anti-inflammatory effect on monocytes than dendrimers terminating with carboxylate groups.^87–89^ The immunomodulatory effect has been proposed to arise from the 3D conformation of the dendrimer.^90^ Analogously, the chemically modified NFCs can be considered as polyvalent molecules due to having numerous charged groups on the cellulose fibrils. A suitable conformation can potentially promote polyvalent interactions where multiple sites on a single polymeric molecule interact with multiple receptors on the cell surface, leading to their clustering and activation of immunoregulatory pathways.^91^ The major advantage of using chemically modified NFCs in immunology models rather than dendrimers or other macromolecules is the inherent gel-formation ability of NFCs, enabling 3D culture, as well as the natural abundance of the cellulose polysaccharide.

The highlight of the pho-NFC-based 3D culture system is that M2-like macrophages could be generated without the addition of external cytokines. Hence, the NFC-based monocyte/macrophage culture system bypasses the need to use expensive reagents, such as M-CSF and IL-4, that are commonly utilized to polarize M2 macrophages *in vitro*.^2^ The significance of the tissue environment in macrophage polarization rather than individual cytokines has also been shown in dermal macrophages whose CD206 expression *in vivo* does not depend on IL-4.^92^ In addition to inducing CD206 expression, pho-NFC could also modulate the cytokine secretion profile of the cells. The cells cultured in pho-NFC secreted anti-inflammatory IL-10 as well as proinflammatory TNF-α and IL-1β at higher levels than the 2D cultured cells. Notably, LPS stimulation of 2D cultured cells was not sufficient to upregulate IL-1β secretion, likely due to the absence of cues required to trigger the proteolytic processing of pro-IL-1β into the mature, secreted form.^93^ In the canonical pathway, pro-IL-1β is cleaved by caspase-1 following the assembly of NLRP3-inflammasome, which can be activated through various mechanisms.^93^ While previous studies investigating inflammasome activation by unmodified NFCs have yielded variable results,^94,95^ the ability of pho-NFC to potentiate IL-1β release demonstrates its immunomodulatory effect on monocyte-derived macrophages.

Despite increased IL-1β secretion, we do not consider pho-NFCs to have caused a proinflammatory M1 response, as M2-like macrophages may also secrete proinflammatory cytokines under certain conditions.^64^ Rather, the changes in secretory activities together with a significant increase in M2-associated CD206 expression suggest that the cells in pho-NFC were transitioning toward a different subtype of M2 macrophages than those in 2D culture. Acquiring diverse macrophage phenotypes *in vitro* is crucial for appreciating macrophage heterogeneity and phenotype regulation *in vivo*. Further research is needed to functionally classify the specific types of immunosuppressive macrophages modeled by the pho-NFC gel. In the future, the environment provided by pho-NFC may be utilized in more complex cultures and cocultures, for example, by incorporating cancer cells and immune cells. Such studies are expected to broaden our understanding of macrophage plasticity along the M1-M2 spectrum and whether the phenotype of macrophages in these models correlates with the function of TAMs. The model could then be utilized for exploring strategies for overcoming the immunosuppressive phenotype of TAMs. Moreover, we anticipate that the cues guiding macrophage differentiation in 3D NFC gels might be generalized into applications beyond immuno-oncology, for instance, for the design of immuno-optimized implants.

While the exact molecular mechanism leading to macrophage polarization in NFC gels is beyond the scope of this study, the findings open intriguing directions for future research. Particularly, the interaction of NFCs with immune cell receptors is a largely unexplored topic, and further research is warranted to tune NFC bioactivity. One potential approach is to vary the degree of phosphorylation to modify the interaction with cells. This will likely require careful optimization of the reaction conditions, as excessive surface modification may alter NFCs in ways that are detrimental to cells.^76,77^ On the other hand, insufficient surface modification may hamper the nanofibrillation process.^24^ If a specific receptor interaction is desired, NFC gels can also be functionalized with specific proteins to modulate the interaction with cells.^31^ Tuning the gel stiffness by adjusting the NFC concentration or by incorporating other polymers may also affect macrophage polarization within the NFC gels. Moreover, the distinct PBMC gene expression profiles observed in the NanoString screening imply that NFC gels may have broad immunomodulatory activities. Therefore, future studies should also explore how the NFC gels influence the phenotype of different immune cell types.

In summary, we have demonstrated the dependence of the macrophage phenotype on NFC chemical modification, challenging the prevalent view of NFC gels as bioinert materials.^96,97^ We therefore argue for using chemically modified NFC gels instead of unmodified NFC gels to develop tools to study macrophage phenotypes in 3D cell culture models. In addition, chemical modification resulted in material properties more advantageous than those of unmodified NFC, such as increased optical transparency and gelation at lower solid content, while maintaining similar mechanical properties. Among the various modification possibilities, TEMPO-mediated oxidation has been a common modification strategy of NFC in biomedical applications.^98–100^ In contrast, incorporation of phosphorus into cellulose initially gained attention as means to improve biomineralization of bone replacement scaffolds.^77,101^ Our findings indicate that phosphorylated NFC gels also represent emergent materials for 3D cell culture applications. Moreover, phosphorylation is envisioned as a more eco-friendly and cost-effective approach to modify NFC than TEMPO-mediated oxidation.^102^ This supports exploitation of phosphorylated NFC gels for further applications, for example, as components in biomaterials for tissue engineering or drug delivery. The shear-thinning property of NFC makes the material also amenable to automated handling, including 3D bioprinting.^103^ Ultimately, fine-tuning NFC properties will enable the use of chemically modified NFC gels in yet unexplored fields of biomedicine.

## 5 Conclusions

Macrophages play a crucial role in the regulation of immune responses, influencing, for example, the resolution of inflammation, the effectiveness of cancer immunotherapies, and the integration of implants. Therein, the modulation of macrophage phenotypes could allow new treatment modalities. However, the currently used 2D culture methods limit our understanding of how to control the macrophage behavior inside tissue-like environments. 3D hydrogel scaffolds are thus necessitated for modeling diverse macrophage phenotypes and functions *in vitro*. Here, we have established the potential of plant-derived 3D NFC gels and their physicochemical properties for modulating the generation of M2-like macrophages from human monocytes. As negatively charged polysaccharides are known to have various immunomodulatory activities, we compared cell responses to carboxylate-, phosphate-, and sulfate-modified NFCs as well as to chemically unmodified NFC. The 3D culture environment provided by the phosphorylated NFC gel offered the most robust platform for promoting the M2-like macrophage phenotype, contrasting with the unmodified NFC gel that was unsuitable for this purpose. These findings contribute to the development of animal-free, surface-tuned 3D culture platforms for *in vitro* modeling of macrophage phenotypes for biomedicine and therapeutic discovery.

## Supplementary Material



## Notes

Distributions of NFC fibril diameters; distributions of the NFC ζ-potentials; viscoelastic properties of NFC gels; light microscopy images of monocyte-derived cells; gating strategy used in flow cytometry analysis; flow cytometry histograms of CD14, CD206, and CD163 expression in different NFC gels; immunofluorescence confocal microscopy of CD14 and CD206 expression; flow cytometry histograms of CD14, CD206, and CD163 expression in IL-4-stimulated cells compared to unstimulated cells and cells in the pho-NFC gel; SDS-PAGE of proteins adsorbed onto NFCs; optical density of 50–70 kDa protein bands in SDS-PAGE; and immunofluorescence confocal microscopy of cell clusters in 3D pho-NFC (PDF)

## Abbreviations

2D: two-dimensional
3D: three-dimensional
CNF: cellulose nanofibril
ECM: extracellular matrix
ELISA: enzyme-linked immunosorbent assay
FBS: fetal bovine serum
IL-1β: interleukin-1β
IL-4: interleukin-4
IL-10: interleukin-10
LPS: lipopolysaccharide
M-CSF: macrophage colony-stimulating factor
MFI: median fluorescence intensity
NFC: nanofibrillar cellulose
PBMC: peripheral blood mononuclear cell
PBS: phosphate-buffered saline
PRR: pattern recognition receptor
PS: protein supplement
SDS-PAGE: sodium dodecyl sulfate polyacrylamide gel electrophoresis
SEM: scanning electron microscopy
TAM: tumor-associated macrophage
TEMPO: 2,2,6,6-tetramethylpiperidine-1-oxyl radical
TGF-β1: transforming growth factor β1
TNF-α: tumor necrosis factor α
