# Supplementary material for: Nanofibrillar Cellulose Hydrogels with Anionic Surface Modifications for Modulating Macrophage Phenotype in 3D Culture
Source: ACS Appl Mater Interfaces. 2025 Jul 3;17(28):40082–100. doi: 10.1021/acsami.5c06549 (PMC12278226; doi:10.1021/acsami.5c06549)
Supplement: Supplementary file 1 [file am5c06549_si_001.pdf]

SUPPORTING INFORMATION

Nanofibrillar Cellulose Hydrogels with Anionic Surface Modifications for Modulating Macrophage Phenotype in 3D Culture

Maria Heilala [1], Rita Turpin [2], Nikolaos Pahimanolis [3], Nonappa [4], Olli Ikkala [1]‡, Juha Klefström [2,5,6,7]‡, Pauliina M. Munne [2]‡**

[1] Department of Applied Physics, Aalto University. P.O. Box 15100, FI-00076 Aalto, Espoo, Finland

[2] Cancer Cell Circuitry Laboratory, Translational Cancer Medicine, Medical Faculty, University of Helsinki. P.O. Box 63 (Haartmaninkatu 8), FI-00014 University of Helsinki, Helsinki, Finland

[3] Oy Keskuslaboratorio-Centrallaboratorium Ab, Tekniikantie 2, FI-02150, Espoo, Finland

[4] Faculty of Engineering and Natural Sciences, Tampere University. P.O. Box 541, FI-33720 Tampere, Finland

[5] Finnish Cancer Institute, FI-00290, Helsinki, Finland

[6] FICAN South, Helsinki University Hospital, FI-00290 HUS, Helsinki, Finland

[7] Department of Cell & Tissue Biology, University of California, San Francisco, 513 Parnassus Avenue, UCSF Campus Box 0512, San Francisco, CA 94143, United States

‡ senior authors with equal contribution

* corresponding authors: pauliina.munne@helsinki.fi, olli.ikkala@aalto.fi

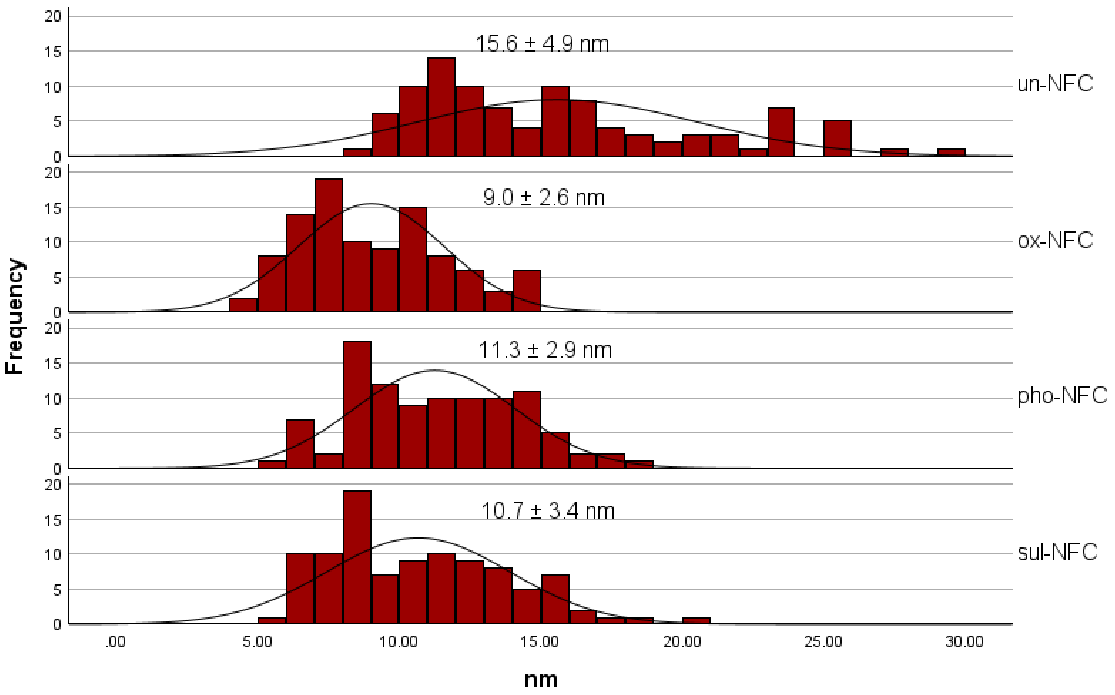

Figure S1. Distributions of NFC fibril diameters. The diameters were estimated from critical point dried samples using SEM. The average fibril diameter and standard deviation are reported for each NFC gel (n = 100 counted fibrils in total, from two independent SEM specimens).

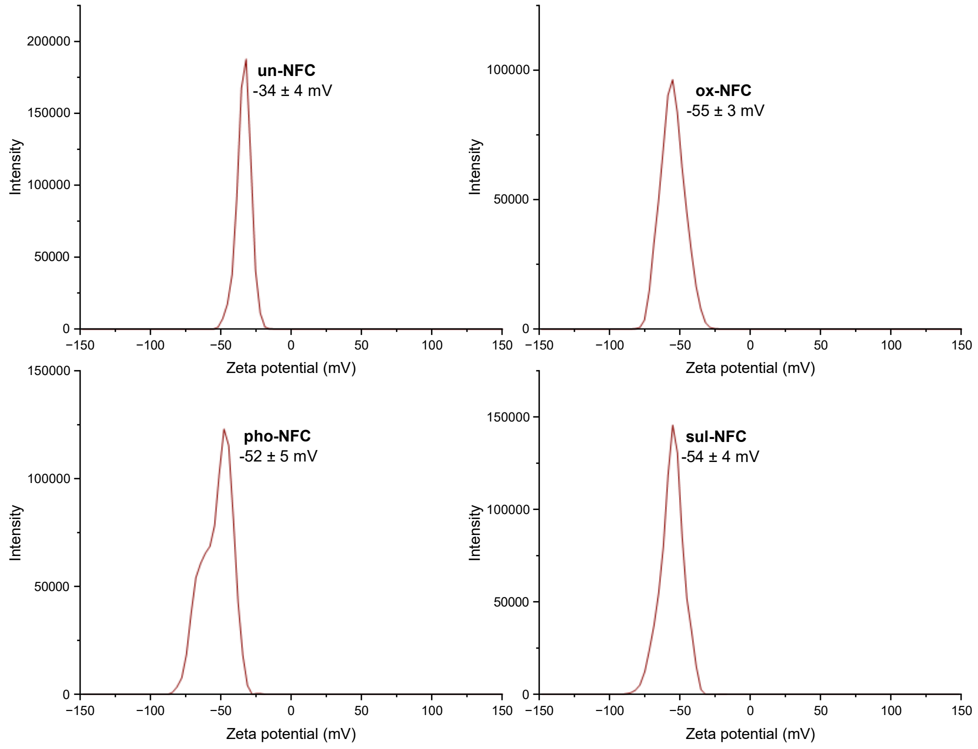

Figure S2. Distributions of NFC ζ-potentials. The ζ-potential indicates the surface charge of 0.01% nanofibrils in water. Before the measurement, samples were sonicated to minimize aggregation. The average ζ-potential and standard deviation are reported for each type of NFC (n = 3 measurements, with 3 runs each).

Table S1. Viscoelastic properties of NFC gels measured by oscillatory shear rheology. Shear storage modulus (*G′*), shear loss modulus (*G″*) and phase angle (δ) for each sample was calculated from the time sweeps performed at 1% strain and 10 rad/s angular frequency at 37 °C. The results represent average values and standard deviations (n = 3 independent measurements, except 1.5% un-NFC where n = 2).

**Gel**	**Dilution**	**Shear storage modulus G′ (Pa)**	**Shear loss modulus G″ (Pa)**	**Phase angle δ (delta degrees)**
1.5% un-NFC	Stock	427.8 ± 5.9	52.4 ± 1.2	7.0 ± 0.07
1.125% un-NFC	Diluted with H_2O	149.6 ± 32.9	20.7 ± 4.4	7.89 ± 0.10
1% ox-NFC	Stock	150.4 ± 3.0	17.4 ± 1.6	6.59 ± 0.46
0.5% pho-NFC	Stock	120.2 ± 6.8	10.2 ± 0.2	4.87 ± 0.38
1% sul-NFC	Stock	365.0 ± 51.4	25.0 ± 2.4	3.94 ± 0.18
0.75% sul-NFC	Diluted with H_2O	161.5 ± 11.2	15.1 ± 1.1	5.36 ± 0.03

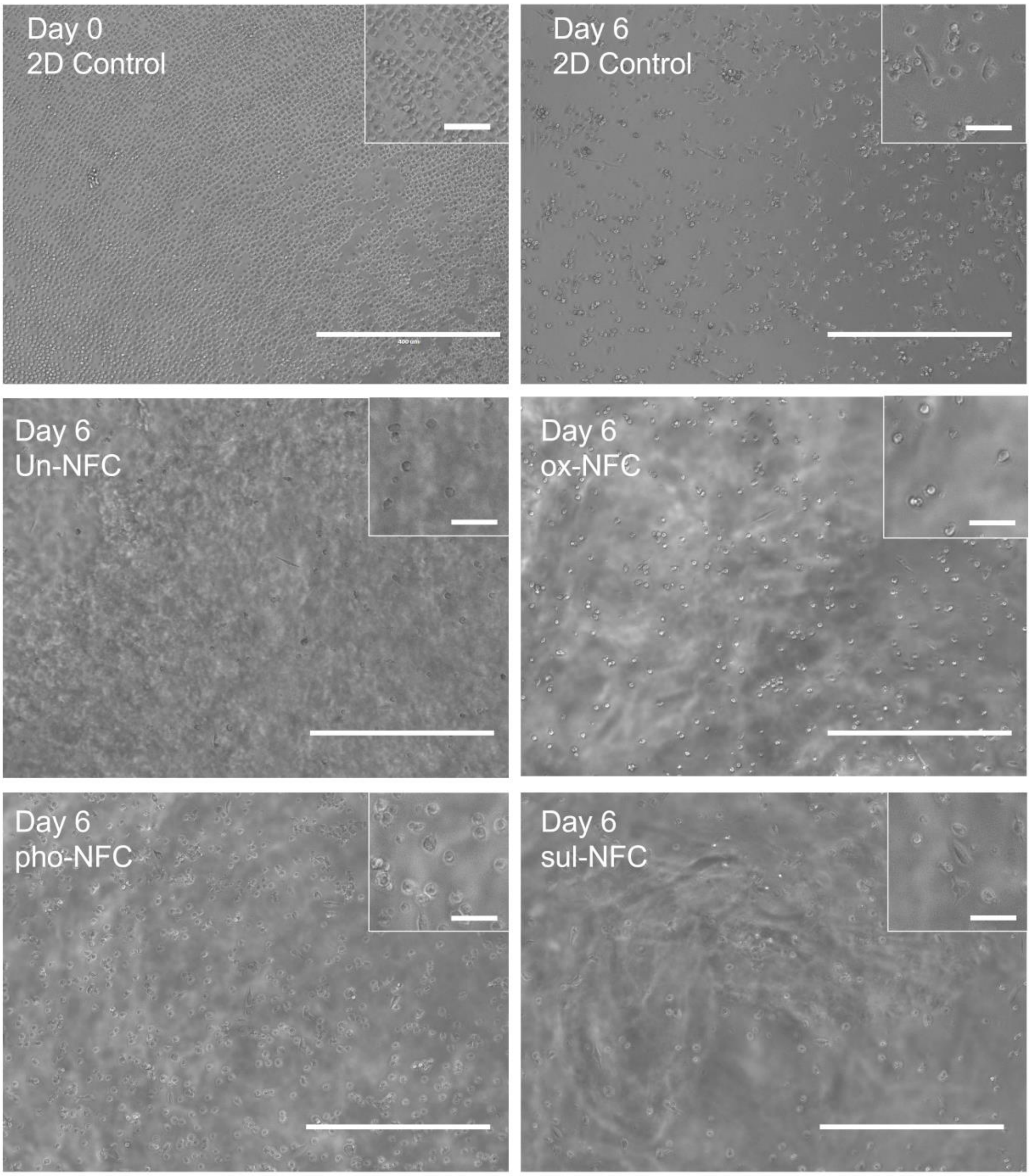

Figure S3. Monocyte-derived cells in different conditions observed with light microscopy. Cells were cultured for 6 days on 2D tissue culture plate (control) or in 3D NFC gels. Scale bar in the overview image is 400 µm, and 50 µm in the inset.

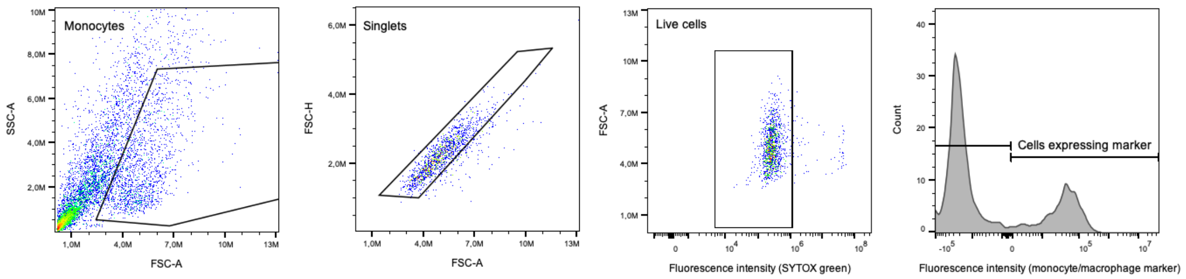

Figure S4. Gating strategy used in flow cytometry analysis.

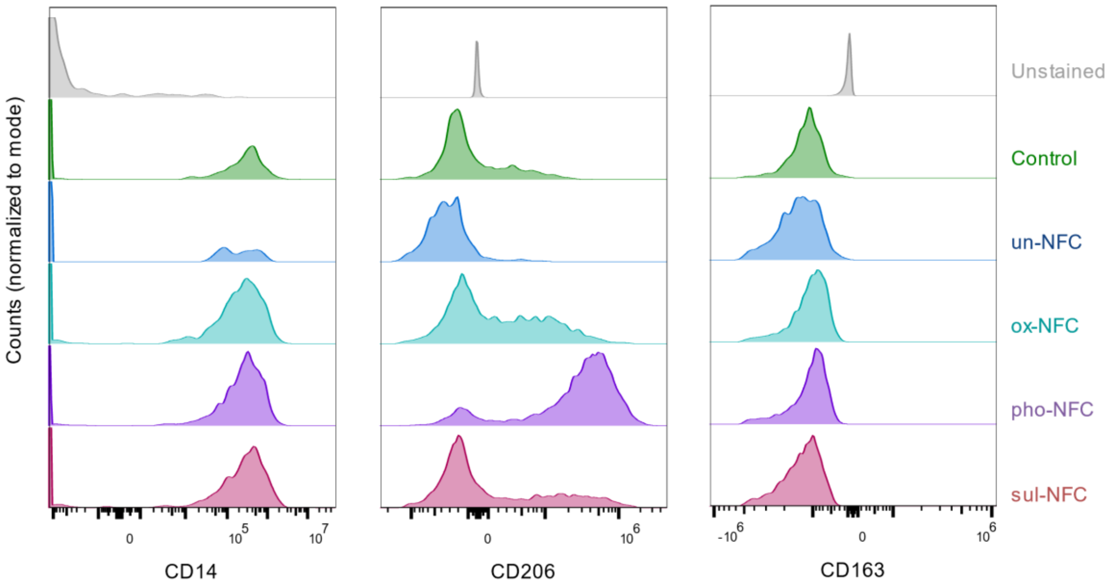

Figure S5. Representative flow cytometry histograms of CD14, CD206 and CD163 expression in different NFC gels. Monocytes were cultured for 6 days on 2D tissue culture plate (control) or in 3D NFC gels. Colored histograms depict fluorescently labelled cells, whereas grey histograms are unlabelled cells (*n* = cells from 5 donors).

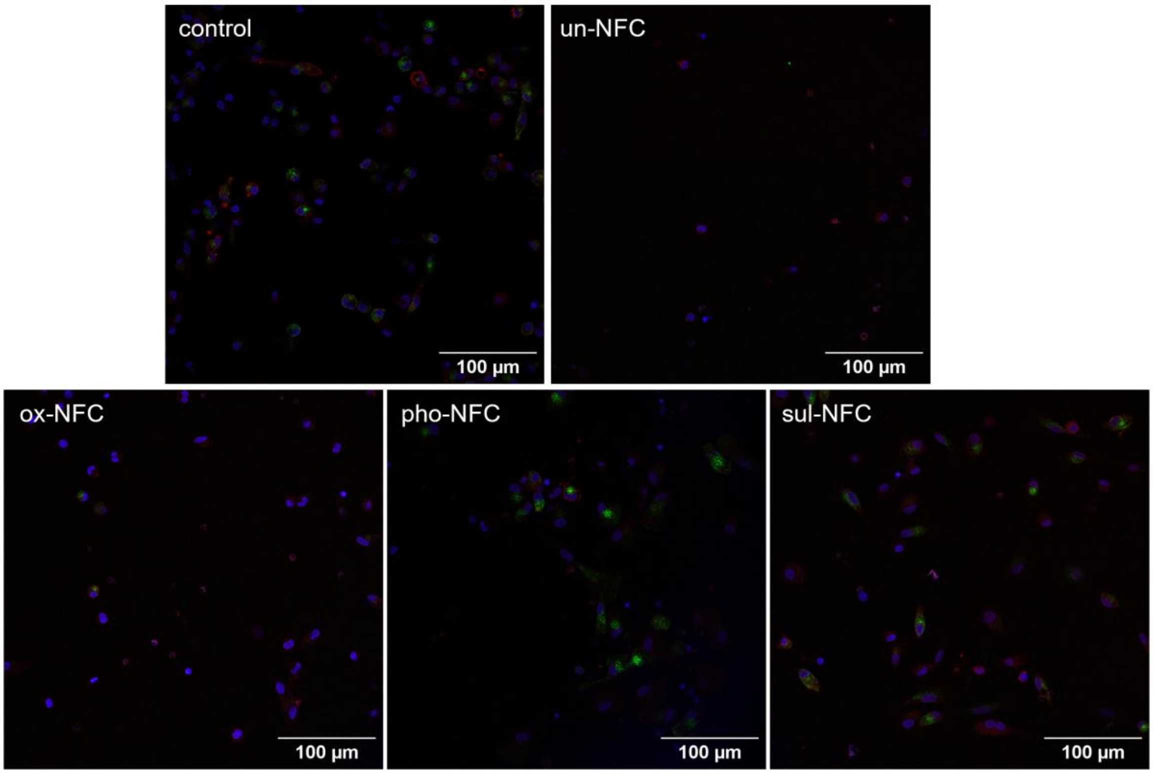

Figure S6. Immunofluorescence confocal microscopy of CD14 and CD206 expression. Monocytes were cultured for 6 days on 2D tissue culture plate (control) or in 3D NFC gels. Representative overlay image of cells from one donor showing staining for CD206 (green), CD14 (red), and nuclei (blue). Scale bar is 100 µm. (*n* = cells from 4 donors).

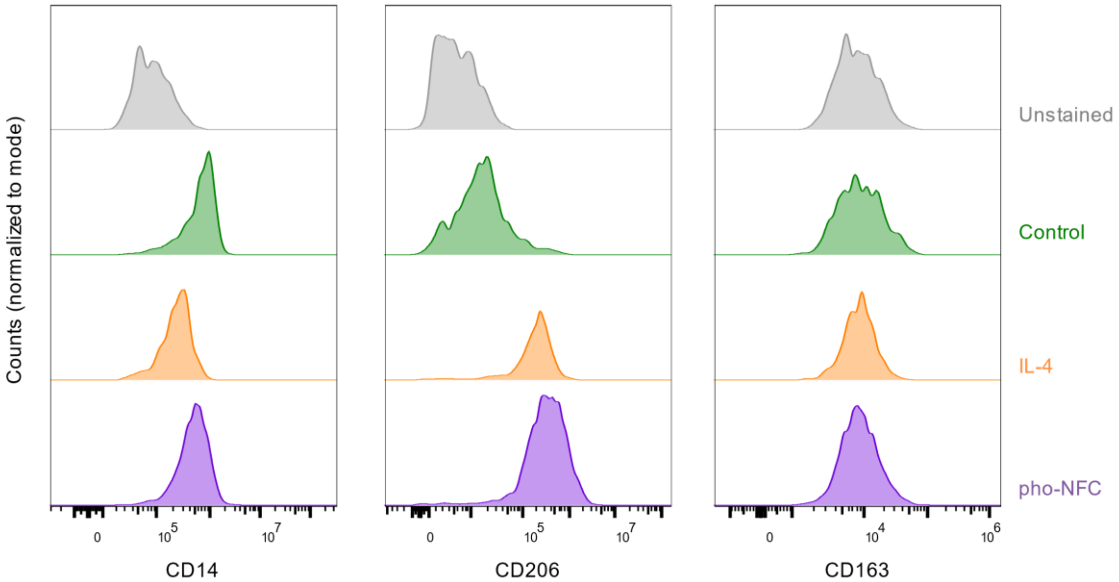

Figure S7. Representative flow cytometry histograms of CD14, CD206 and CD163 expression in IL-4 stimulated cells compared to unstimulated cells and cells in pho-NFC gel. Monocytes were cultured for 6 days on 2D tissue culture plate (control, IL-4) or in 3D pho-NFC gel. For IL-4 stimulation, cells were cultured in the presence of M-CSF and treated with IL-4 on day 3. Cells in control and pho-NFC received no treatment. Colored histograms depict fluorescently labelled cells, whereas grey histograms are unlabeled cells (*n* = cells from 5 donors).

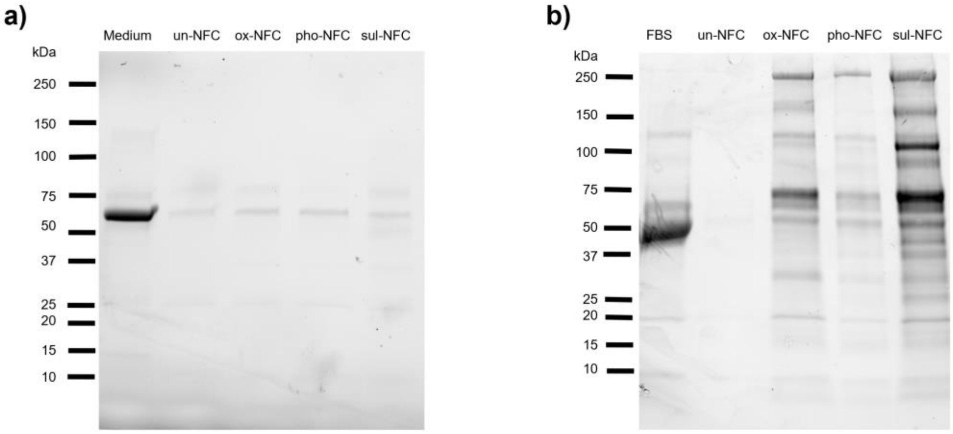

Figure S8. SDS-PAGE of protein adsorption onto NFCs. Representative SDS-PAGE of 0.5% NFCs incubated with **a)** Mammocult medium with 10% serum-free protein supplement (*n* = 3), and **b)** with 100% FBS (*n* = 3). Before electrophoretic separation, weakly adsorbed proteins were removed by washing the NFCs repeatedly with PBS.

Table S2. Optical density (OD) of strongly adsorbed proteins on 0.5% NFCs. The results represent average values and standard deviations measured with ImageJ (*n* = 3).

Gel	OD of band at 70 kDa	OD of band at 50 kDa	Ratio of upper/lower band
un-NFC	55 ± 11	21 ± 5	2.2 ± 1.0
ox-NFC	115 ± 16	54 ± 11	1.9 ± 0.3
pho-NFC	23 ± 5	46 ± 31	0.7 ± 0.1
sul-NFC	106 ± 11	106 ± 38	1.1 ± 0.3

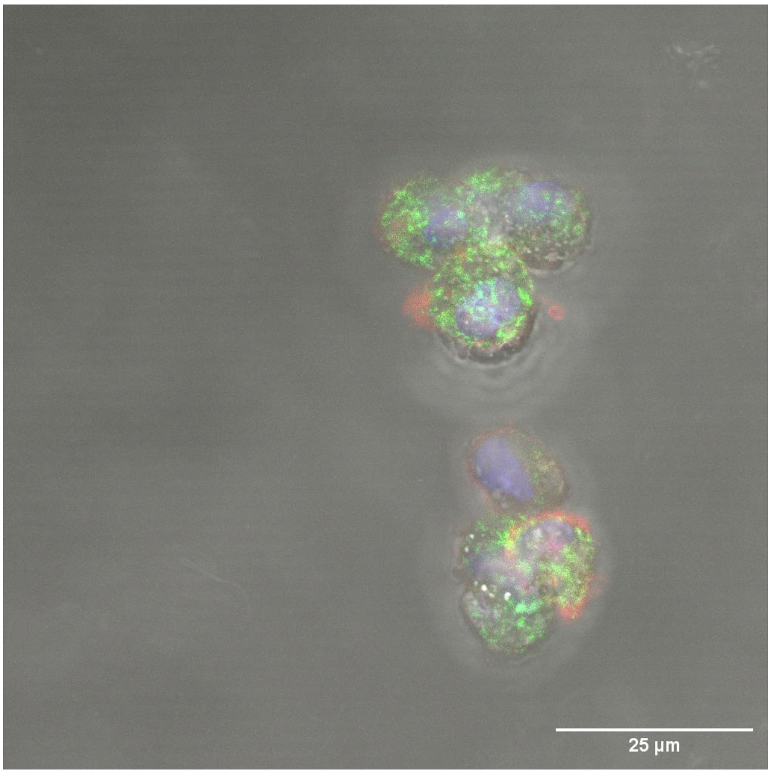

Figure S9. Immunofluorescence confocal microscopy of cell clusters in 3D pho-NFC. Representative overlay image of a multicellular cluster formed after 6 days in culture. The maximum intensity projection shows staining for CD206 (green), CD14 (red), and nuclei (blue) throughout the cluster (*n* = cells from 5 donors). Scale bar is 25 µm.
